# A nicotine-induced positive feedback loop between HIF1A and YAP1 contributes to epithelial-to-mesenchymal transition in pancreatic ductal adenocarcinoma

**DOI:** 10.1186/s13046-020-01689-6

**Published:** 2020-09-07

**Authors:** Qiwen Ben, Wei An, Yunwei Sun, Aihua Qian, Jun Liu, Duowu Zou, Yaozong Yuan

**Affiliations:** 1 Department of Gastroenterology, Ruijin Hospital, Shanghai Jiaotong University School of Medicine, 197 Ruijin Er Road, Shanghai, 200025 PR China; 2 Department of Gastroenterology, Changhai Hospital of Second Military Medical University, 168 Changhai Road, Shanghai, 200433 China; 3 Department of Oncology, Shanghai General Hospital, Shanghai Jiao Tong University School of Medicine, Shanghai, PR China

**Keywords:** Pancreatic ductal adenocarcinoma, YAP1, Nicotine, HIF1A, Epithelial-to-mesenchymal transition

## Abstract

**Background:**

Nicotine, an active ingredient in tobacco, can promote epithelial-to-mesenchymal transition (EMT) processes that enhance the aggressiveness of a number of human cancers. In the present study, we investigated whether cigarette smoke/nicotine drives EMT in pancreatic ductal adenocarcinoma (PDAC).

**Methods:**

Quantitative real-time PCR, western blot, immunohistochemistry, and immunofluorescence assays were used to evaluate Yes-associated protein 1 (YAP1) expression associated with cigarette smoking in human PDAC tissue samples and with nicotine exposure in PDAC cell lines. Bioinformatics, loss- and gain- of- function experiments, luciferase reporter assays, chromatin immunoprecipitation (ChIP), and murine tumor xenograft models were performed to examine the function of YAP1 in PDAC and to identify potential mechanisms of action.

**Results:**

Exposure to smoking or nicotine promoted EMT and tumor growth in PDAC cells and in xenograft tumors. Functional studies revealed that YAP1 might drive nicotine-stimulated EMT and oncogenic activity in vitro and in vivo. In human PDAC tissues, upregulation of YAP1 was associated with “ever smoking” status and poor overall survival. In term of mechanism, hypoxia inducible factor (HIF)1A promoted YAP1 nuclear localization and YAP1 transactivation by directly binding to the hypoxia responsive elements of the YAP1 promoter upon nicotine treatment. Nicotine stimulated HIF1A and YAP1 expression by activating cholinergic receptor nicotinic alpha7 (CHRNA7). In addition, YAP1 increased and sustained the protein stability of HIF1A.

**Conclusions:**

These data demonstrate that YAP1 enhances nicotine-stimulated EMT and tumor progression of PDAC through a HIF1A/YAP1 positive feedback loop. Developing inhibitors that specifically target YAP1 may provide a novel therapeutic approach to suppress PDAC growth, especially in PDAC patients who have a history of smoking.

## Background

Pancreatic ductal adenocarcinoma (PDAC), the main type of pancreatic cancer, is a malignant solid tumor that has one of the highest mortality rates [1]. Currently, curative treatment for PDAC is surgery, but more than 80% of patients are diagnosed at advanced stages and present with an unresectable tumor mass [2]. The survival of patients with metastatic PDAC remains dismal, with a median survival of less than 1 year [1]. Cigarette smoking is related to PDAC incidence and clinical outcomes [3, 4]. Nicotine (Nic), an active ingredient in tobacco, has been reported to drive tumorigenesis and accelerate metastasis by activating cholinergic receptor nicotinic alphas (CHRNAs), of which alpha 7 subunit (CHRNA7) has been shown to be the primary receptor in PDAC [5–7].

The epithelial-to-mesenchymal transition (EMT) plays a critical role in PDAC progression and metastasis [8, 9]. During the EMT process, tumor cells change from an epithelioid morphology to a mesenchymal morphology, and exhibit decreases in epithelial markers, such as E-cadherin (E-cad) and claudin-1 [10], and increases in mesenchymal markers, such as vimentin (Vim) and n-cadherin (N-cad), as well as EMT- related transcription factors, such as Twist1/2, the Slug/Snail family, and ZEB1/2 [9]. Nic can stimulate EMT and increase the aggressiveness of many types of cancer [11, 12]. To date, it is unclear if Nic-induced EMT phenotypes occurs in the development of PDAC, and the mechanisms by which Nic may contribute to EMT and progression in PDAC are not completely known.

The Hippo- yes-associated protein (YAP) pathway plays a vital part in modulating metabolism, organ-size, and tumorigenesis [13, 14]. YAP1 is a transcriptional co-activator of the Hippo pathway, which can decrease YAP1 activity by promoting cytoplasmic localization of YAP1 [15]. In many human malignancies, upregulation of YAP1 is reported to be associated with enhanced cell growth, tumor formation, and worse clinical outcomes [13, 16]. The phosphorylation status and localization of YAP1 is the most important mechanism regulating YAP1 function [17]. Recent studies have demonstrated a link between Nic exposure and YAP1 activity in non-small cell lung cancer (NSCLC) and esophageal squamous cell cancer (ESCC) [18, 19]. However, the potential regulatory mechanism of YAP1 in nicotine-induced PDAC progression is unknown.

Hypoxia is an important feature in the microenvironment of solid tumors, especially in PDAC, due to their avascular morphology. Members of the hypoxia inducible factor (HIF) family of transcription factors, including *HIF1A* and *HIF2A*, drive tumor cells towards an invasive phenotype [20, 21]. Smoking or Nic exposure is reported to promote HIF1A expression in human nasopharyngeal carcinoma cells [22, 23]. Nevertheless, whether Nic can induce changes in HIF1A expression or activity in PDAC remains unclear.

In the present study, we demonstrate that YAP1 plays a critical role in nicotine-induced EMT in PDAC, both in vitro and *vivo*. Importantly, we show that HIF1A promotes YAP1 nuclear localization by inhibiting YAP1 phosphorylation, and can subsequently enhance YAP1 transactivation by directly binding to the hypoxia response elements (HRE) upon Nic treatment. In addition, YAP1 can increase and sustain HIF1A protein stability, thereby establishing a HIF1A/YAP1 positive feedback loop that mediates Nic-stimulated EMT and tumor progression in PDAC.

## Materials and methods

### Cell culture and human sample collection

The human PDAC cell lines, Panc-1 and BxPC-3, were obtained from the Cell Bank of the Chinese Academy of Sciences (Shanghai, China). The cell lines were authenticated by analysis of short tandem repeats, and were confirmed to be free of mycoplasma contamination at the beginning of this study. All cell lines were used for experiments within 30 passages from thawing, and were maintained in Dulbecco’s modified Eagle medium (DMEM; Gibco) supplemented with 10% FBS at 37 °C in a humidified atmosphere of 95% air and 5% CO_2_.

A total of 173 sequential formalin-fixed paraffin-embedded (FFPE) PDAC tissue samples were available from cases who had received radical surgery at the Changhai Hospital (Shanghai, China). All samples with tumor tissues (TTs) and matched adjacent non-cancerous tissues (ANTs) were used to construct a tissue microarray (TMA). No patients had a history of chemotherapy or radiotherapy prior to surgery. Pathological diagnosis was assessed according to the 7th edition of the American Joint Committee on Cancer (AJCC) staging system [24]. Retrospective clinicopathologic data were also obtained from these patients. Participants were defined as “ever-smokers” if they had smoked more than 100 cigarettes during their lifetime. Accordingly, “never smokers” were determined if they smoked less than 100 cigarettes during their lifetime. The use of these specimens and patient information was approved by the Ethics Committee of at the Changhai Hospital (Shanghai, China) in accordance with recognized ethical guidelines of Declaration of Helsinki.

### Immunohistochemistry (IHC) and immunofluorescence (IF)

For IHC assessment of YAP1, HIF1A, E-cad, and Vim in human PDAC tissues, the DAKO Envision system (Dako, Carpinteria, California, USA) was used, as described previously [25]. Briefly, after paraffin-embedded sections of tumor tissues were heated, the sections were incubated with primary antibodies (Supplementary Table s1) overnight at 4 °C. A DAB substrate kit (Dako) was used to perform the chromogenic reaction. IHC staining scores for YAP1, HIF1A, and E-cad were obtained by multiplying the intensity (0, negative; 1, low; 2, medium; and 3, high) with the extent of staining (0, 0%; 1, 0–10%; 2, 10–50%; 3, 50–75%; 4, > 75%). The final scores were used to classify the samples into three grades: 0–3, weak staining (+); 4–6, medium staining (++); and 7–12, strong staining (+++). For subsequent analyses, weak and medium staining were grouped together as a low expression (score 0–6), and strong staining was considered as high expression (score 7–12). For Vim evaluation, the IHC staining score was classified by the area of positive-staining stroma into four categories: score 0 (negative, < 25%), score 1 (focal, 25–50%), score 2 (multifocal, 50–75%), or score 3 (diffused staining, 75–100%). Two pathologists, who were blinded to the clinical data, independently scored all sections. For evaluation of Ki67, the number of positive cells was calculated in three representative areas of high staining.

IF staining was performed on PDAC cell lines and tissue samples. Briefly, cells were fixed in 4% polyformaldehyde, washed in PBS, and blocked with 5% BSA in PBS. Then, these cells or frozen sections were incubated with primary antibodies against E-cad (1:100), Vim (1:200), and YAP1 (1:100) at 4 °C overnight, and subsequently incubated with fluorescent dye–labeled secondary antibodies at room temperature for one hour. DAPI (1:1000) was used to counterstain cell nuclei and images were captured with a confocal fluorescence microscope.

### Gene microarray analysis

We treated Panc-1 cells with Nic (1.0 μM) or DMSO for 24 h. Total RNA was extracted from the cells and analyzed on an Arraystar Human LncRNA Microarray V4.0 Microarray, provided by Kangchen Biotech (Shanghai, China). Cells were prepared and analyzed in three independent biological replicates. Differences between groups were analyzed using the significance analysis of microarrays (SAM) algorithm.

### Preparation of shRNA lentivirus and establishment of stable cell lines

The lentivirus-delivered shRNAs against YAP1 (Lv-shYAP1) and the negative control (Lv-shNC) were acquired from Shanghai GeneChem (Shanghai, China). The viral particles and the establishment of shYAP1 stable clones in Panc-1 cells were performed according to the manufacturer’s instructions [26]. The control clone (shNC) was constructed similarly. The transfection efficiency was confirmed by immunoblotting. Of the two stable cell lines, we selected the cell line exhibiting the most efficient knockdown of YAP1 for the xenograft tumor models.

### Plasmids and siRNAs

The expression plasmids, pcDNA4-YAP1 and pcDNA3.0-HIF1A, were purchased from Addgene (Boston, USA). For knockdown studies, siRNA targeting human YAP1 (siYAP1) and HIF1A (siHIF1A) were synthesized. The targeting sequences are shown in Supplementary Table s2. Non-targeting siRNAs (siNCs) and negative control empty vectors (EVs) were used as controls for the siRNA and expression plasmids, respectively. Plasmids or siRNAs were transiently transfected into PDAC cells using Lipofectamine 2000 (Invitrogen, Carlsbad, CA, USA), according to the manufacturer’s instructions.

### Quantitative RT-PCR

Total RNA was prepared from fresh PDAC tissues and cell lines using TRIzol reagent (Invitrogen, Carlsbad, CA, USA), according to the manufacturer’s instructions. qRT-PCR was conducted in a 96-well real time PCR system (Bio-Rad Inc., Hercules, CA) using a SYBR® Premix Ex Taq kit (Takara Bio Inc., Shiga, Japan). GAPDH was used to normalize gene expression. The primers are shown in Supplementary Table s2. Relative gene expression was calculated from the qRT-PCR data using the 2 ^−ΔΔCT^ method.

### Protein extraction and western blot analysis

Total protein was extracted from cells and tissue using RIPA buffer containing a mixture of protease inhibitors. Equal amounts of total protein (30 μg) were subjected to SDS- polyacrylamide gel separation, followed by overnight incubation at 4 °C with primary antibodies against: YAP1, pYAP1, HIF1A, E-cad, Vim, N-cad, Claudin-1, MST1, LATS1, CHRNA3, CHRNA5, CHRNA7, and GAPDH. Anti-mouse IgG and anti-rabbit IgG HRP-conjugated secondary antibodies were used. Protein expression levels were quantified by normalizing to the GAPDH band using image J software (National Institutes of Health, Bethesda, MD, USA).

### Subcellular fractionation assay

A Nuclear and Cytoplasmic Protein Extraction Kit (Beyotime, Shanghai, China) was used to separate PDAC cells cytoplasmic and nuclear extracts, according to the manufacturer instructions. Briefly, cells were washed in cold PBS and lysed with Cyt buffer. After centrifugation at 800×*g* for 5 min at 4 °C, cytosolic supernatants were collected, and purified nuclei were resuspended in SDS-sample buffer as the nuclear fraction. Samples were immunoblotted for YAP1 expression. GAPDH and Histone H3 were used as cytoplasmic and nuclear markers, respectively.

### Construction of the truncated YAP1 regulatory regions and luciferase reporter assay

The HREs in the YAP1 promoter were predicted using the JASPAR database (http://jaspar. genereg.net/). Genomic DNA was extracted from Panc-1 cells following the manufacturer’s instructions, and the different truncated mutant YAP1 regulatory regions were amplified by PCR. The primers used to amplify the truncated YAP1 promoter regions are shown in Supplementary Table s2. YAP1 promoter region fragments were inserted into the pGL3 vector (Promega, Madison, WI), as described previously [26]. The YAP1 promoter reporter plasmids were co-transfected with HIF1A, or empty vector (EV) into cells. For the HIF1A transcriptional activity assay, pGL4-HREs-luciferase plasmid (4 μg) and pRL-TK plasmid (4 μg) were incubated with PDAC cells. These cells were also incubated with 1.0 μm Nic or DMSO. After 24 h, luciferase activity was detected using the Dual-luciferase reporter assay system (Promega, Madison, WI).

### Chromatin immunoprecipitation (ChIP)

For ChIP assay, PDAC cells were treated with 1.0 μm Nic or DMSO for 12 h, as described previously [27]. Briefly, protein-DNA complexes were produced by adding 1% formaldehyde to the cells, and the chromatin was sheared by sonication to a mean fragment size of 300–500 bp. Cells were immunoprecipitated overnight with an anti-HIF1A antibody or rabbit IgG, and the associated genomic DNA was assessed by PCR and agarose gel electrophoresis. The specific primers for putative HREs in the YAP1 promoter are shown in Supplementary Table s2.

### Cell viability, migration and transwell assays

To evaluate cell proliferation rates, MTT assay was used. Briefly, cells were seeded in 96-well plates at 2 × 10^3^ cells/well and incubated with DMSO or 1.0 μm Nic. Sample absorbance at 490 nm was evaluated on a microplate spectrophotometer (Thermo, Spectronic, Madison, WI, USA). For the cell scratch–wound assays, cells were cultured in six-well plates until confluent, and horizontal streaks were created in the cells using the a 20-μL pipette tip. Then, cells were washed and incubated with DMSO or 1.0 μm Nic. An inverted microscope was used to measure the migratory distance at 0 h and 24 h, and cell migration was assessed by measuring gap sizes in multiple fields. For transwell assays, cells (1.0 × 10 [5]/ml) were placed in the top side of transwell chambers (8 μm pore size membranes, Millipore) with matrigel for invasion. Vehicle (DMSO) or nicotine was added into the upper well for 24 h. The invaded cells were fixed, stained and counted in five random fields.

### Mouse xenograft model

All animal studies were performed following the Institutional Animal Care and Use Committee of Shanghai Jiaotong University (Shanghai, China). Six-week-old male BALB/c mice were obtained from Shanghai SLAC Laboratory Animal Center (Shanghai, China). All animals were maintained in a barrier facility in high-efficiency particulate air–filtered racks. Logarithmic phase Panc-1 cells (5.0 × 10 [6]/100 μL) transfected with Lv-shYAP1 or control vector were inoculated subcutaneously into the dorsal flank of mice. Tumor volume was evaluated by the following formula: volume (mm^3^) = length × width × height × 0.52. When tumor volume reached 75–125 mm^3^, mice were randomized into three groups, and that day was defined as day 1. Nicotine or DMSO was administered intraperitoneally thrice weekly for 3 weeks. On day 22, all mice were euthanized, and the tumors were excised and weighed.

### Bioinformatics and statistical analysis

Multiple databases, including GEPIA (http://gepia.cancer-pku.cn/) [28], StarBase 3.0 (http://starbase.sysu.edu.cn/) [29], and KM plotter (http://kmplot.com/analysis), were queried for gene expression in PDAC tissues. Data are shown as mean ± SD. Differences between groups were evaluated using unpaired t-test for two groups or the chi square test. Survival analyses were performed using the Kaplan-Meier method with the log-rank test and univariate and multivariate Cox regression. All statistical analyses were performed using the PASW Statistics 19.0 software program (SPSS, Chicago, IL, USA). A two tailed *p*-value of *P* < 0.05 was considered to be statistically significant. In the graphed data *, **, and *** denote *p* values of *P <* 0.05, *P* < 0.01, and *P* < 0.001, respectively.

## Results

### Smoking/nic-induced EMT is accompanied by increased YAP1 expression in PDAC

Our previous research indicated that nicotine induces an EMT process in PDAC cells [30]. Here we examined whether there is an association between smoking status and expression of EMT markers in human PDAC tissues. Using IHC assays, we found that expression levels of E-cad were significantly lower, while expression levels of Vim were higher, in PDAC tumor tissue (TT) samples from patients who were “ever smokers” than from patients who were “never smokers” (Supplementary Fig. s1a and b). A similar difference was also observed in adjacent non-tumor (ANT) pancreas samples (Supplementary Fig. s1c and d). These results were further confirmed by IF assays in human PDAC tissues (Supplementary Fig. s1e and f). Treatment of Panc-1 cells with 1.0 μM Nic in vitro also significantly increased Vim expression, as demonstrated by IF analysis (Supplementary Fig. s1g). Additionally, phase contrast microscopy revealed EMT-like morphological changes in Panc-1 cells treated with 1.0 μM Nic (Supplementary Fig. s1h). These data indicate that smoking/nicotine exposure can stimulate EMT processes in human PDAC cells and tissues.

**Fig. 1:**
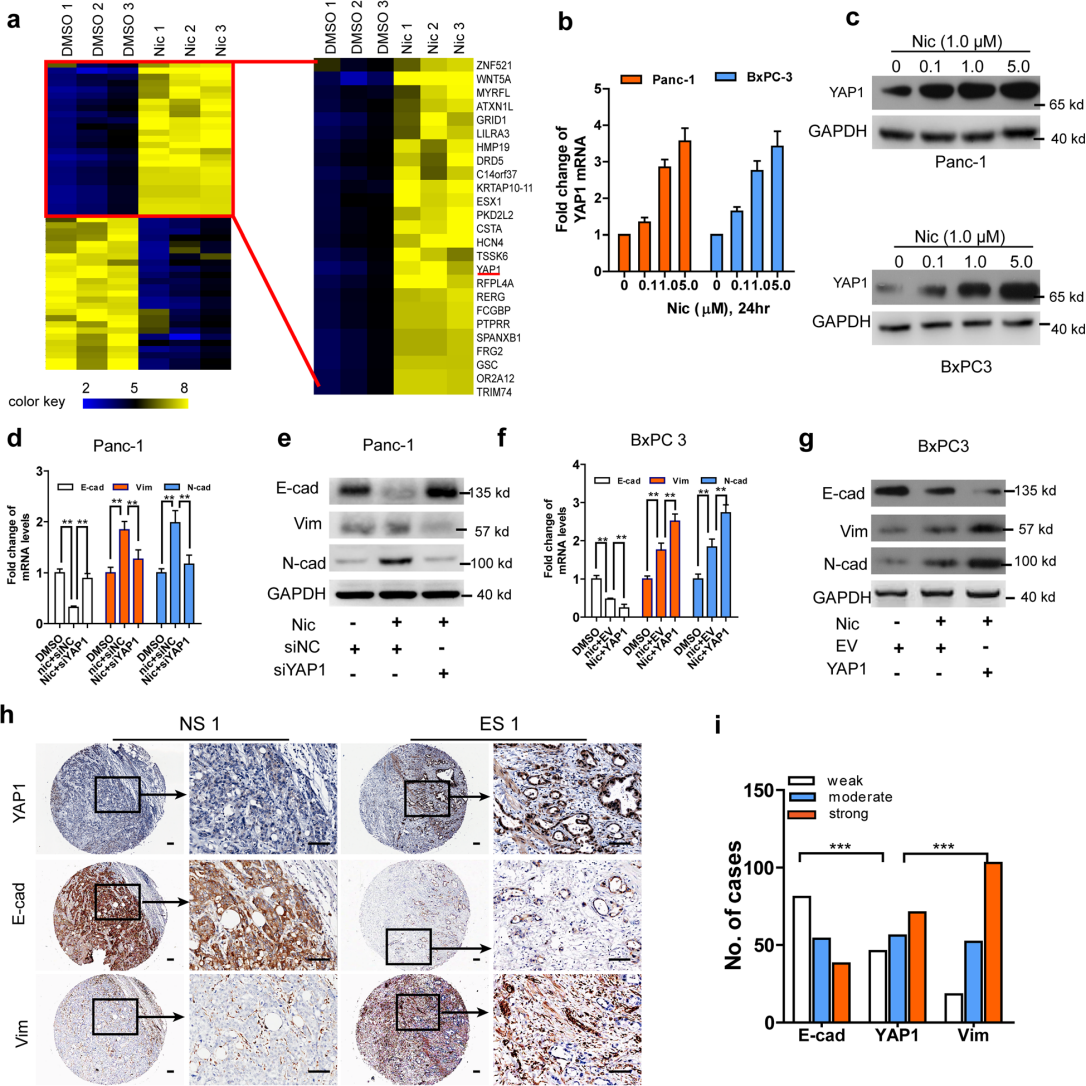
Smoking/nicotine-induced EMT is accompanied by increased YAP1 expression in PDAC cells**. a**, Heatmap to illustrate the hierarchical clustering of the mRNA differential expression profiles in nicotine-treated Panc-1 cells. **b-c**, qRT-PCR and western blot assays of YAP1 mRNA (**b**) and protein (**c**) expression levels in Panc-1 and BxPC-3 cells after treatment with different concentrations (0, 0.1,1.0, 5.0 μM; 24 h) of Nic. **d-g**, qRT-PCR and western blot assays of the expression levels of EMT markers (E-cad, Vim and N-cad) in PDAC cells stimulated with DMSO or Nic (1.0 μM, 24 h) after transfection with shYAP1 in Panc-1 cells (**d-e**) or overexpression vectors in BxPC3 cells (**f-g**). **h**, Immunohistochemical staining and quantitative results for YAP1, E-cad and Vim in human PDAC tissue microarray from ESs and NSs (*n* = 173). Scale bar, 100 μm. EMT, epithelial-mesenchymal transition; TT, tumor tissues; ANT, adjacent non-cancerous tissues; ES, ever smoker; NS, never smoker; E-cad, E-cadherin; Vim, vimentin; Nic, nicotine; PDAC, pancreatic ductal adenocarcinoma. GAPDH was used as an internal reference. Data are shown as the mean ± SD of three replicates. Chi square test was used for statistical analysis. **P < 0.01, ***P < 0.001

To investigate gene expression altered by nicotine exposure in Nic-treated PDAC cells, we screened differential mRNA expression profiles of Panc-1 cells treated with nicotine or DMSO by high-throughput microarray. Following 1.0 μM Nic treatment for 24 h, 118 mRNAs were found to be dysregulated (fold change ≥2.0, data not shown). The top 25 upregulated and downregulated mRNAs, are illustrated in a heat map (Fig. 1a). The YAP1 gene, which has been reported to enhance PDAC progression, was upregulated 5.31-fold in nicotine-treated cells. Subsequently, we evaluated the effects of treatment with nicotine on YAP1 expression in Panc-1 and BxPC-3 cells, and found that YAP1 mRNA (Fig. 1b) and protein (Fig. 1c) were significantly upregulated by exposure to nicotine in a dose-dependent manner, as compared to DMSO-treated cells. We knocked down YAP1 in two PDAC cell lines with two YAP1-targeting siRNAs. The siYAP1#2 siRNA exhibited higher knockdown efficiency (Supplementary Fig. s2a-b), and was used to perform subsequent siRNA-mediated YAP1 knockdown experiments. We also overexpressed YAP1 in two PDAC cell lines (Supplementary Fig. s2c-d). Cell proliferation, migration, and invasion induced by nicotine was significantly attenuated in YAP1-silenced PDAC cells, while YAP1 overexpression strengthened these effects (Supplementary Fig. s2e-j). In addition, treatment with nicotine led to an enhanced EMT process, which was attenuated by YAP1 knockdown and strengthened by YAP1 overexpression **(**Fig. 1d-g**)**. We further used IHC to assess the expression levels of YAP1, E-cad, and Vim in human PDAC tissue samples stratified according to smoking status. We found that “ever smokers” with PDAC had higher expression levels of YAP1 in both TT and ANT pancreas samples, compared with “never smokers” (Supplementary Fig. s3 a-b). In addition, expression of YAP1 was inversely correlated with E-cad in cancer specimens (Spearman’s *r =* − 0.238, *P* < 0.001), but was positively associated with Vim expression (Spearman’s *r =* 0.219, *P <* 0.001; Fig. 1h-i). Furthermore, we discovered that YAP1 was positively correlated with the majority of EMT markers in PDAC tissues from the cancer genome atlas (TCGA) dataset (Supplementary Fig. s4 a-h). Based on these data, we hypothesized that YAP1 may play an important role in regulating nicotine-induced EMT in PDAC.

### Knockdown of YAP1 inhibits nicotine-induced tumor growth and EMT of PDAC in mice

**Fig. 2:**
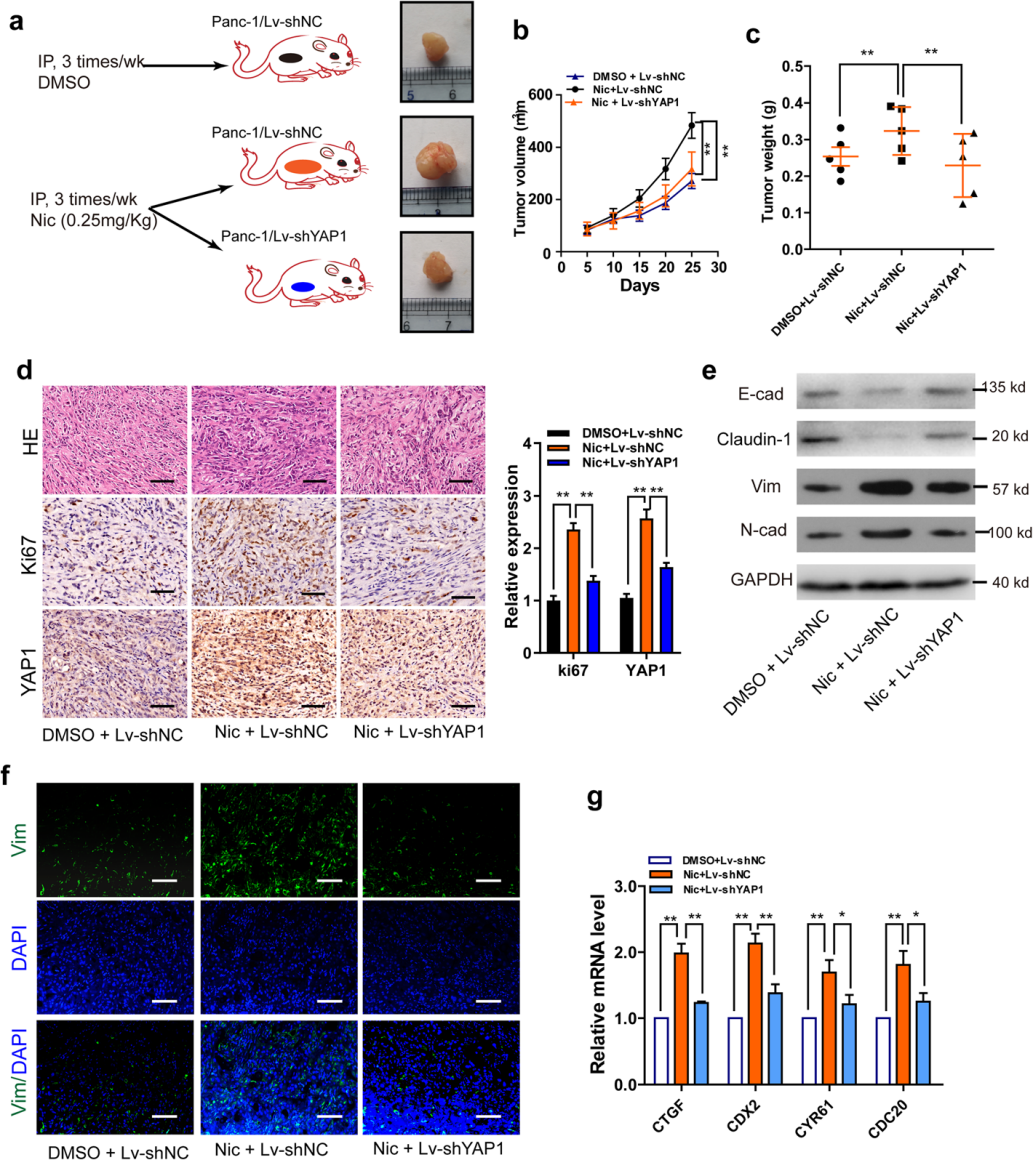
Knockdown of YAP1 inhibits nicotine- induced tumor growth and EMT in a xenograft model of PDAC in mice. **a**, Diagram depicts the overall xenograft study; **b-c**, The volumes (**b**) and weights (**c**) of xenograft tumors from mice in the three experimental groups. **d**, HE and Ki67 staining in xenograft tumor in the three experimental groups. Scale bar, 100 μm. **e**, Western blotting was conducted to examine E-cad, Vim, N-cad expression in the three experimental groups. **f**, Immunofluorescence assays of the expression levels of Vim in xenograft tumor in the three experimental groups. Scale bar, 100 μm. **g,** qRT-PCR assays of the expression levels of YAP target genes, CTGF, CDX2, CYR61, and CDC20 in mice xenograft tumors. EMT, epithelial-mesenchymal transition; HE, Hematoxylin and eosin, E-cad, E-cadherin; Vim, vimentin; N-cad, N-cadherin; NC, negative control; Nic, nicotine. GAPDH was used as an internal reference. Data are shown as the mean ± SD of three replicates.*P < 0.05; **P < 0.01

Fifteen nude mice challenged with subcutaneous pancreatic xenograft tumors were randomized into three groups (Fig. 2a**)**. The animal experiments demonstrated that YAP1 knockdown inhibited nicotine-enhanced tumor growth, and reduced expression of Ki67 and YAP1 (*P <* 0.01; Fig. 2b-d). Immunoblot and IF assays on the xenograft tumors revealed that YAP1 knockdown significantly decreased nicotine-promoted EMT processes **(**Fig. 2e-f**)**. As an important transcriptional co-activator, YAP1 can change the expression of various target genes, including CTGF, CDX2, CYR61, and CDC20 [17]. We found that knockdown of YAP1 significantly inhibited nicotine-stimulated upregulation of CTGF, CDX2, CYR61, and CDC20 **(**Fig. 2g). These data demonstrate that YAP1 may mediate the tumor-promoting role of nicotine in PDAC in vivo.

### Expression levels of YAP1 are associated with progressive clinical characteristics and poor overall survival of PDAC patients

**Fig. 3:**
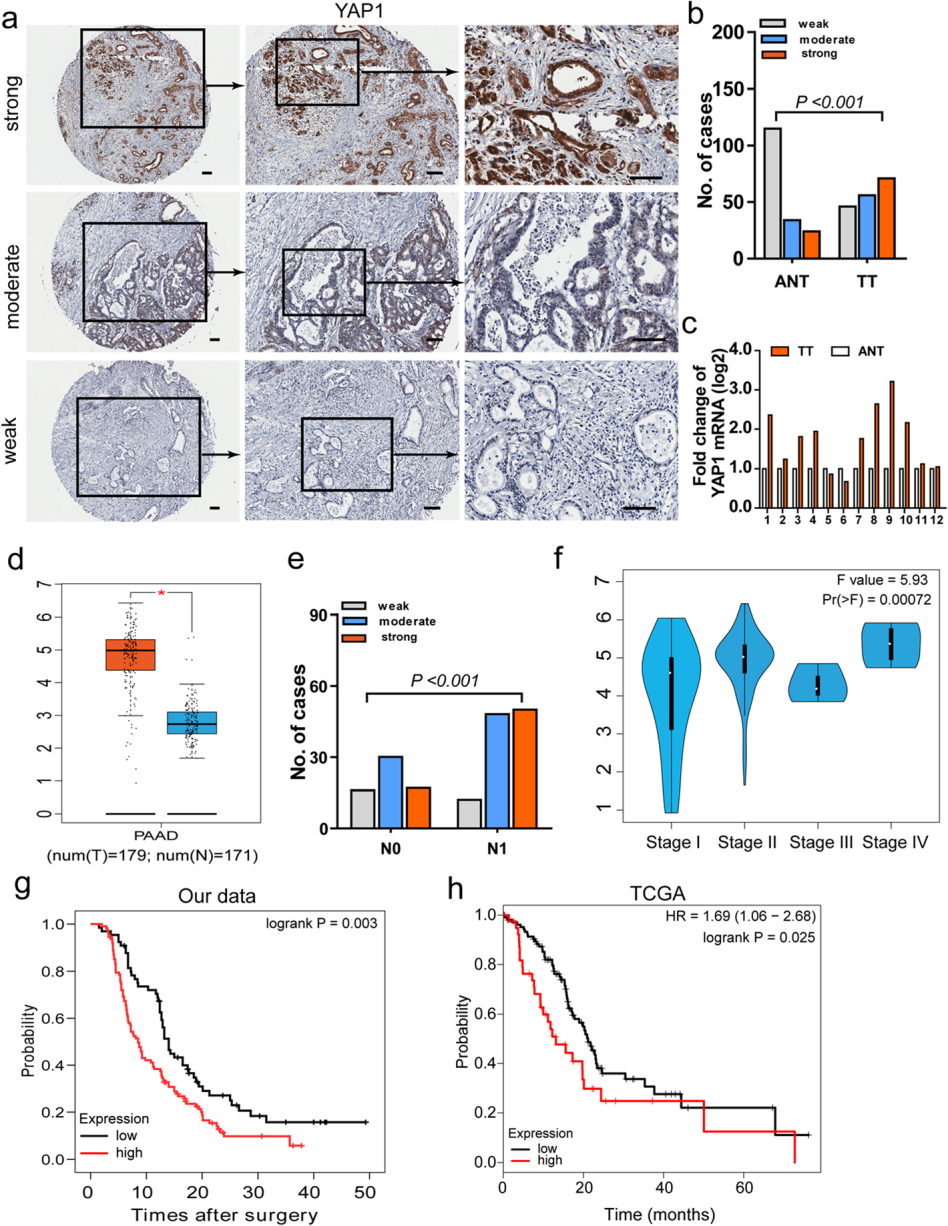
Expression levels of YAP1 are associated with progressive clinical characteristics and poor overall survival of PDAC patients. **a-b,** Representative images and statistical analyses of YAP1 expression in PDAC specimens from our cohort. Scale bars, 100 μm. Chi square test was used for statistical analysis. **c,** qRT-PCR analysis of YAP1 expression in TT and paired ANT specimens obtained from 12 PDAC patients. **d**, Results from TCGA database showed that expression of YAP1 mRNA in TT was significantly higher than that in ANT. Chi square test was used for statistical analysis. **e**, Staining score of YAP1 was positively assocaited with lymph node metastasis. Chi square test was used for statistical analysis. **f-g**, Kaplan-Meier analysis of overall survival of PDAC cases from our cohort (f; Log rank test, *P* = 0.003) and TCGA dataset (g; Log rank test, *P* = 0.025) according to expression of YAP1. Patients were grouped according to high and low YAP1 expression. TT, tumor tissues; ANT, adjacent non-cancerous tissues; PDAC, pancreatic ductal adenocarcinoma. GAPDH was used as an internal reference

We examined the expression of YAP1 in human PDAC (*n* = 173) in a TMA-based IHC study. The clinicopathologic characteristics of the TMAs are shown in Supplementary Table s3. YAP1 protein expression levels in PDAC specimens were much higher than those in ANT specimens (Fig. 3a-b and Supplementary Fig. s5a). qRT-PCR analysis of the mRNA expression levels of YAP1 in paired normal pancreatic tissue and PDAC specimens (*n* = 12) confirmed this finding, which was also confirmed in gene expression analysis from the TCGA (*n* = 179; Fig. 3c-d). We then evaluated the association between YAP1 expression and clinicopathological features in PDAC cases. Although there were no significant associations between YAP1 expression and sex, age, tumor size, T category, and neural invasion (data not shown), chi-square analyses confirmed that higher expression of YAP1 was positively associated with lymph mode invasion (Fig. 3e). Analysis of the data from TCGA validated these findings (Fig. 3f). Additionally, Kaplan-Meier survival analysis from our data and from the TCGA dataset showed that the survival time for patients with low expression levels of YAP1 was significantly longer than patients with high expression levels of YAP1 (Fig. 3g-h). Univariate and multivariate Cox analyses confirmed that YAP1 is an independent predictor of unfavorable OS in PDAC (hazard ratio [HR] = 1.51, 95% confidence interval [CI]: 1.04–2.20; Table 1). These data suggest that YAP1 plays critical roles in PDAC development and progression.

**Table 1:** Univariate and multivariate Cox regression analyses of overall survival in pancreatic ductal adenocarcinoma

Parameters	Univariate analysis, HR (95% CI)*	P	Multivariate analysis, HR (95% CI)	***P*** value
**T stage**				
T3-T4 vs. T1-T2	1.52 (1.03–2.25)	**0.035**	1.43 (1.01–2.02)	**0.043**
**N stage**				
N1 vs. N0	1.53 (1.09–2.15)	**0.013**	1.32 (1.05–1.66)	**0.019**
**Tumor grade**				
G3 vs. G1 + G2	1.19 (0.68–2.09)	0.544		
**Tumor size, cm**				
≥ 4 vs.<4	1.29 (0.93–1.80)	0.133	1.28 (0.92–1.80)	0.145
**Vascular invasion**				
Yes vs. No	0.98 (0.68–1.38)	0.852		
**Adjuvant therapy**				
Yes vs. No	0.63 (0.44–0.90)	**0.012**	0.75 (0.58–0.98)	**0.032**
**Neural invasion**				
Yes vs. No	1.21 (1.01–1.45)	**0.041**	1.26 (1.01–1.57)	**0.038**
**YAP1 staining**				
High vs. Low,	1.67 (1.18–2.36)	**0.004**	1.51 (1.04–2.20)	**0.033**

NOTE: *HR* hazard ratio, *CI* confidence interval

Tumor classification and stage were referred to the 7th edition of UICC on cancer staging system. Bold indicates significance

### Nicotine stimulates YAP1 nuclear translocation

**Fig. 4:**
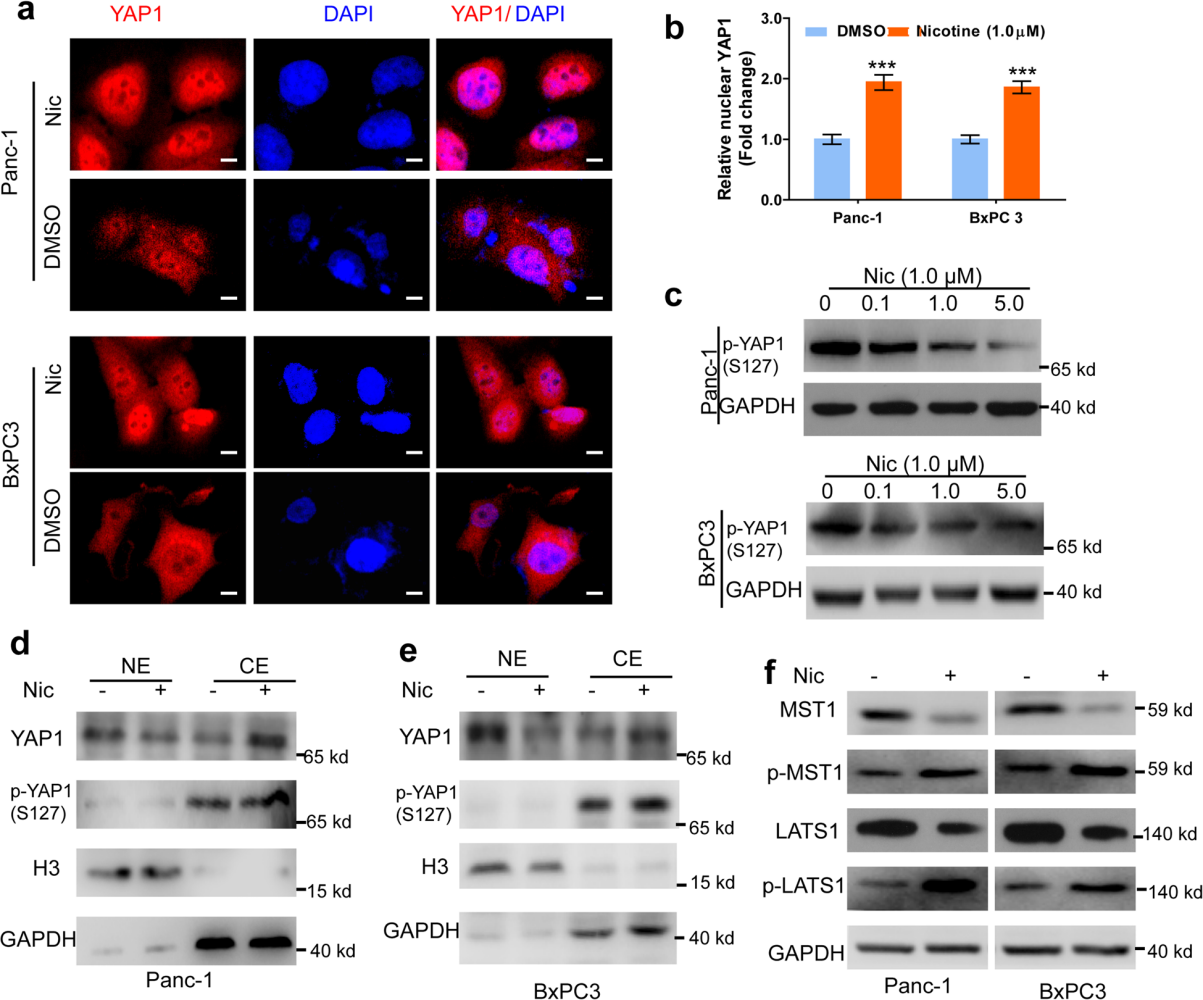
Nicotine stimulates nuclear translocation of YAP1. **a**, Immunofluorescence assay of the localization of YAP in Panc-1 and BxPC3 cells upon nic (1.0 μM) or DMSO treatment for 24 h. Scale bars, 200 μm. **b-c,** Western blot assay of the expression of p-YAP(S127) in Panc-1 (**b**) and BxPC3 (**c**) cells with nicotine treatment at 0, 0.1, 1.0, 5.0 μM for 24 h. **d-e,** Western blot assays of the expression of YAP1 and p-YAP1 in the nuclear or cytoplasmic extracts in Panc-1 (**d**) and BxPC3 (**e**) cells with nic (1.0 μM) or DMSO treatment. Histone H3 and GAPDH were used as internal control for nuclear and cytoplasm, respectively. **f-g,** Western blot assay of the expression of MST1, p-MST1, LATS1 and p-LATS1 in Panc-1 and BxPC3 cells with nic (1.0 μM) or DMSO treatment. GAPDH was used as an internal reference. NE, nuclear extracts; CE, cytoplasmic extracts; Nic, nicotine. Data are shown as the mean ± SD of three independent experiments. *p < 0.05, **p < 0.01, ***p < 0.001

Nuclear localization is essential for the activity of YAP1. We determined whether nicotine treatment affects the subcellular localization of YAP1 in PDAC cells. IHC assessment indicated that YAP1 was localized in both the cytoplasm and nucleus, and mainly in the nucleus (Supplementary Fig. s5b-c). To explore the clinical significance of cytoplasmic and nuclear expression of YAP1, we evaluated associations of cytoplasmic and nuclear YAP1 expression with OS, and we found significantly shorter OS time (*P* < 0.001; Supplementary Fig. s5d) in patients with nuclear, but not cytoplasmic, expression of YAP1 (Supplementary Fig. s5e). Likewise, IF assessment confirmed that nicotine treatment promoted YAP1 nuclear translocation in both BxPC-3 and PANC-1 cells (Fig. 4a). Western blot analysis showed that the expression of YAP1 serine 127 phosphorylation (p-YAP1) was downregulated upon nicotine treatment in a dose-dependent manner (Fig. 4b-c and Supplementary Fig. s6a-c). Furthermore, we evaluated the levels of YAP1 in the cytoplasm and nucleus, and found that nicotine stimulated high YAP1 expression in nucleus and low expression in the cytoplasm, while p-YAP1 was exclusively expressed in the cytoplasm (Fig. 4d-e). The activated kinase cascade MST1/LATS1 is a key mediator that prohibits YAP activity by direct phosphorylation of YAP1, sequestering p-YAP1 in the cytoplasm [31]. We found that nicotine treatment inhibited MST1/LATS1, and increased the protein expression of phosphorylated MST1 and LATS1 in both PDAC cell lines (Fig. 4f and Supplementary Fig. s6d-e). Together, these data indicate that nicotine affects the intranuclear and cytoplasmic distribution of YAP1.

### Nicotine stimulates YAP1 nuclear localization through association with HIF1A expression

**Fig. 5:**
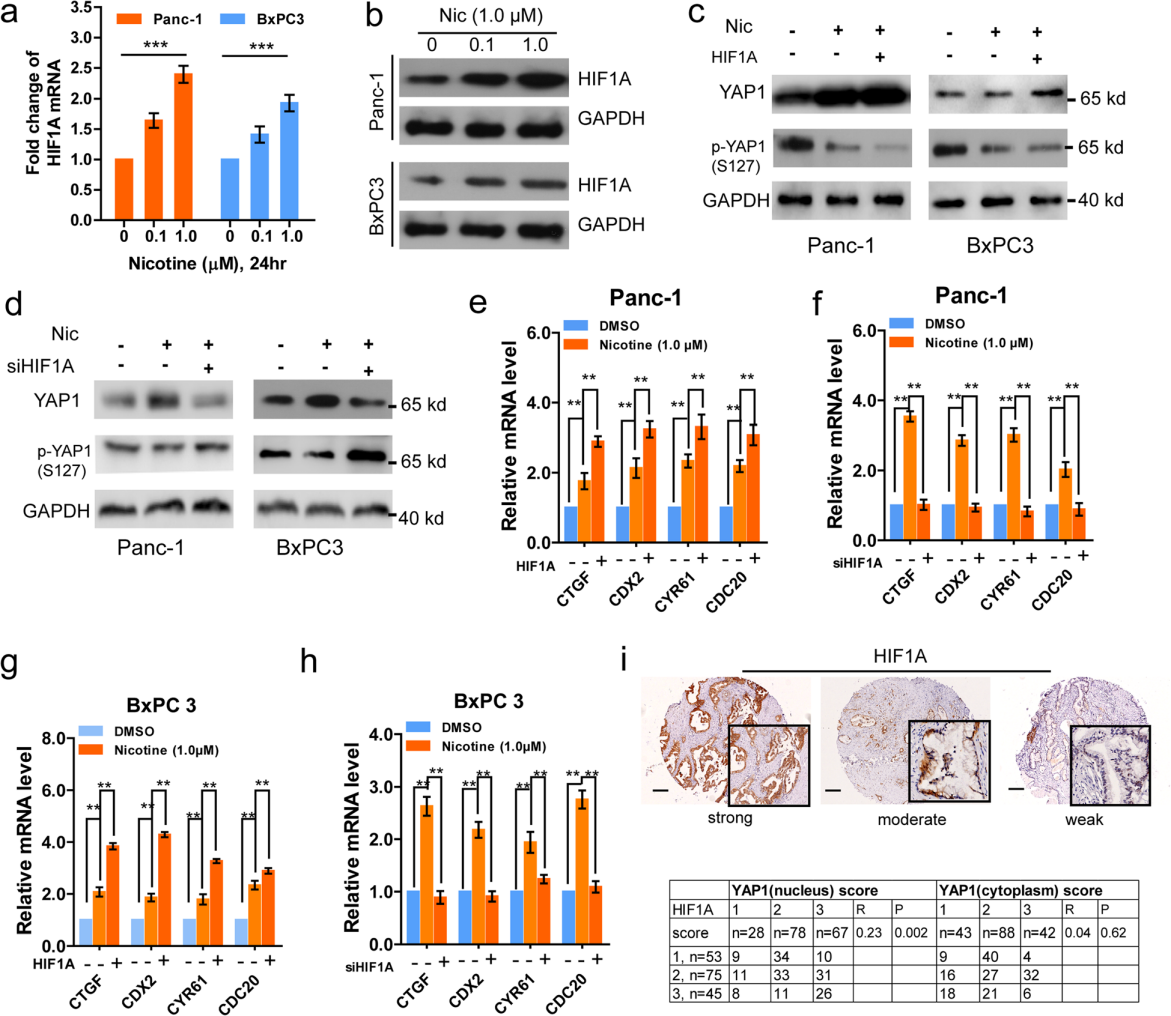
Nicotine stimulates YAP1 nuclear location through association with HIF1A expression. **a-b,** qRT-PCR and western blot assays of HIF1A mRNA (**a**) and protein (**b**) expression levels in Panc-1 and BxPC-3 cells after treatment with different concentrations (0, 0.1,1.0 μM; 24 h) of nicotine. **c-d**, Western blot assays of YAP1, p-YAP1 expression levels in Panc-1 and BxPC-3 cells after transfection with the indicated vectors upon nicotine treatment. **e-h,** qRT-PCR assays of YAP1 target gene, CTGF, CDX2, CYR61, and CDC20, expression levels in Panc-1 (**e-f**) and BxPC-3 (**g-h**) cells after transfection with the indicated vectors upon nicotine treatment. **i,** Representative images of HIF1A expression and its association with YAP cellular localization in PDAC specimens based on IHC assay. Scale bars, 200 μm. *p < 0.05, **p < 0.01, ***p < 0.001

Nicotine can regulate HIF1A expression in several types of cancer, and therefore we suspected that nicotine might affect YAP expression through a HIF1A-mediated mechanism. We confirmed that nicotine promoted the mRNA and protein expression of HIF1A in a dose-dependent manner (Fig. 5 a-b). After HIF1A knockdown or overexpression (Supplementary Fig. s7 a-d), we observed that HIF1A knockdown significantly inhibited, while overexpression of HIF1A enhanced, the proliferation and migration of PDAC cells in the presence of nicotine (Supplementary Fig. s7 e-l). Interestingly, nicotine stimulated total YAP1 expression, but inhibited phosphorylation of YAP1. Overexpression of HIF1A enhanced, whereas knockdown of HIF1A attenuated, nicotine-induced changes in YAP1 expression and phosphorylation in Panc-1 and BxPC3 cell**s** (Fig. 5 c-d). Furthermore, overexpression of HIF1A enhanced, whereas knockdown of HIF1A attenuated, expression of YAP1 target genes (Fig. 5 e-h). Additional IHC analysis revealed that HIF1A staining scores were positively associated with YAP1 nuclear staining, but not cytoplasmic staining (Fig. 5 i). These results demonstrate that nicotine stimulates YAP1 nuclear translocation through a HIF1A-mediated mechanism.

### HIF1A promotes YAP1 transactivation upon nicotine treatment

**Fig. 6:**
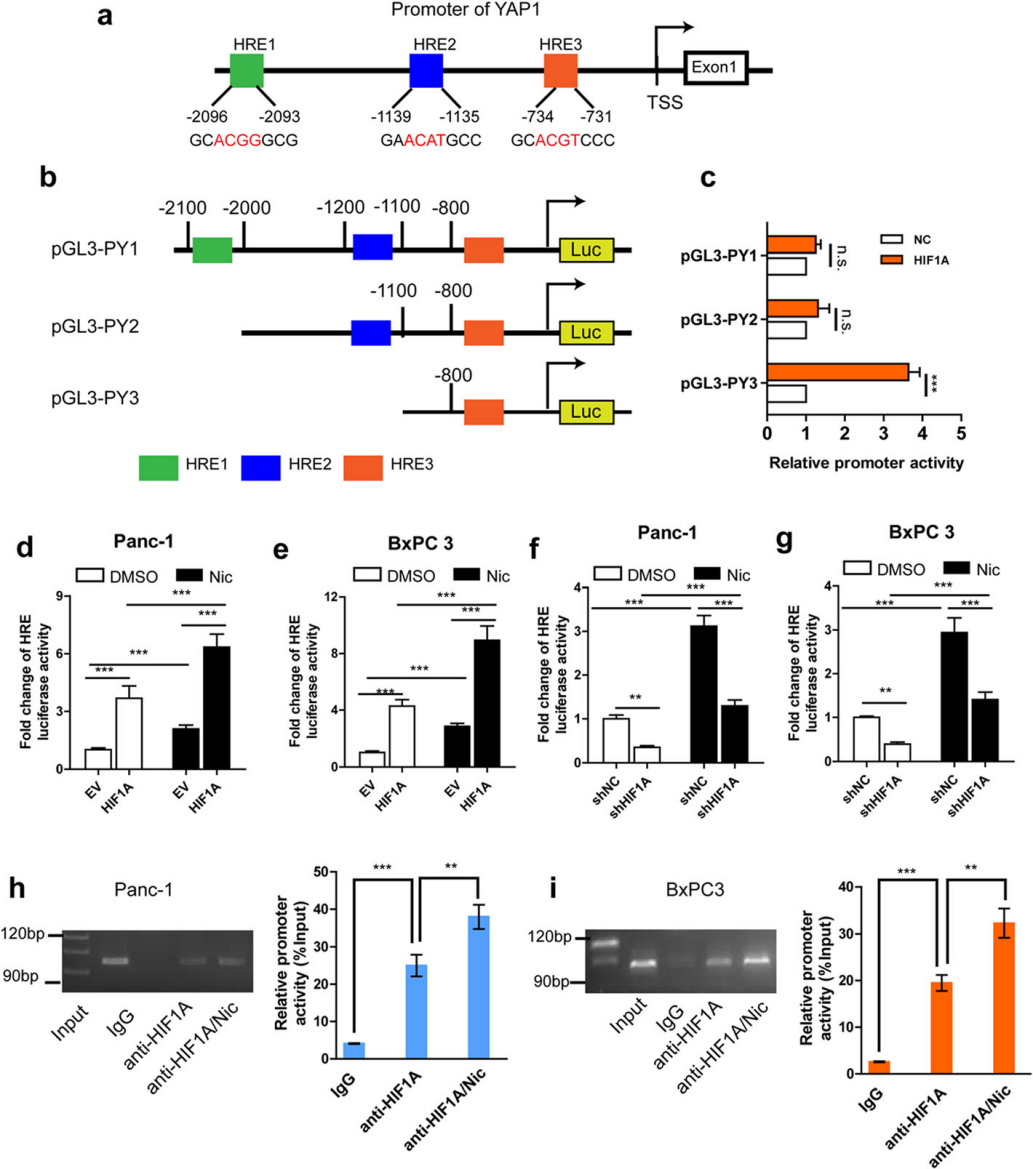
HIF1A promotes YAP1 transactivation upon nicotine treatment. **a,** Three potential HIF1A–binding sites (HRE1–3) in the YAP1 promoter sequence. **b,** The full-length and deletion mutants YAP1 promoter. **c,** Dual luciferase assay of YAP1 promoter activity after cotransfected the deletion mutant reporters with or without HIF1A expression vectors or control vector into Panc-1 cells. **d-g,** Dual luciferase assay of promoter activity in Panc-1 and BxPC3 cells after cotransfection a pGL4-HREs-luciferase plasmid and pRL-TK with HIF1A overexpression (**d-e**) or siRNA (**f-g**) vectors upon nicotine or DMSO treatment. **h-i,** ChIP-RT-PCR assay of the YAP promoter activity in Panc-1 (**h**) and BxPC3 (**i**) cells with or without nicotine treatment. A specific anti-HIF1A antibody was used and IgG was used as a control. The experiments were performed three times independently. HRE, hypoxia response elements; Nic, nicotine; n.s., not significant. *p < 0.05, **p < 0.01, ***p < 0.001

To further analyze the mechanism by which YAP1 expression is regulated by HIF1A upon nicotine treatment, we surveyed the YAP1 promoter sequence and identified three potential HIF1A–binding sites (HRE1–3) upstream to the transcription start site (TSS; Fig. 6a). The full-length YAP1 promoter and deletion mutant reporter constructs were generated, and were then co-transfected with or without HIF1A expression vectors or NC vectors into Panc-1 cells. Dual luciferase assays confirmed that deletion of the region from − 800 to − 700 bp, covering the HRE3 site, significantly attenuated YAP1 promoter activity (Fig. 6b-c). To further evaluate HIF1A transcriptional activity following nicotine treatment, we co-transfected an HRE-luc plasmid along with HIF1A expression vectors or shRNA into PDAC cells. Nicotine stimulated YAP1 promoter activity in PDAC cells (Fig. 6d-g); overexpression of HIF1A enhanced, whereas knockdown of HIF1A attenuated, YAP1 promoter activity. To determine whether HIF1A directly binds to HREs on the YAP promoter, we performed ChIP assays. Immunoprecipitation with an anti-HIF1A antibody pulled down the predicted binding-site sequence of the YAP1 promoter, and nicotine treatment significantly enhanced these effects in both PDAC cells (Fig. 6h-i**)**. These results indicate that, upon nicotine treatment, HIF1A binds to the promoter region of YAP1 and upregulates YAP1 transcription in PDAC cells.

### Nicotine stimulates HIF1A and YAP1 through association with CHRNA7

**Fig. 7:**
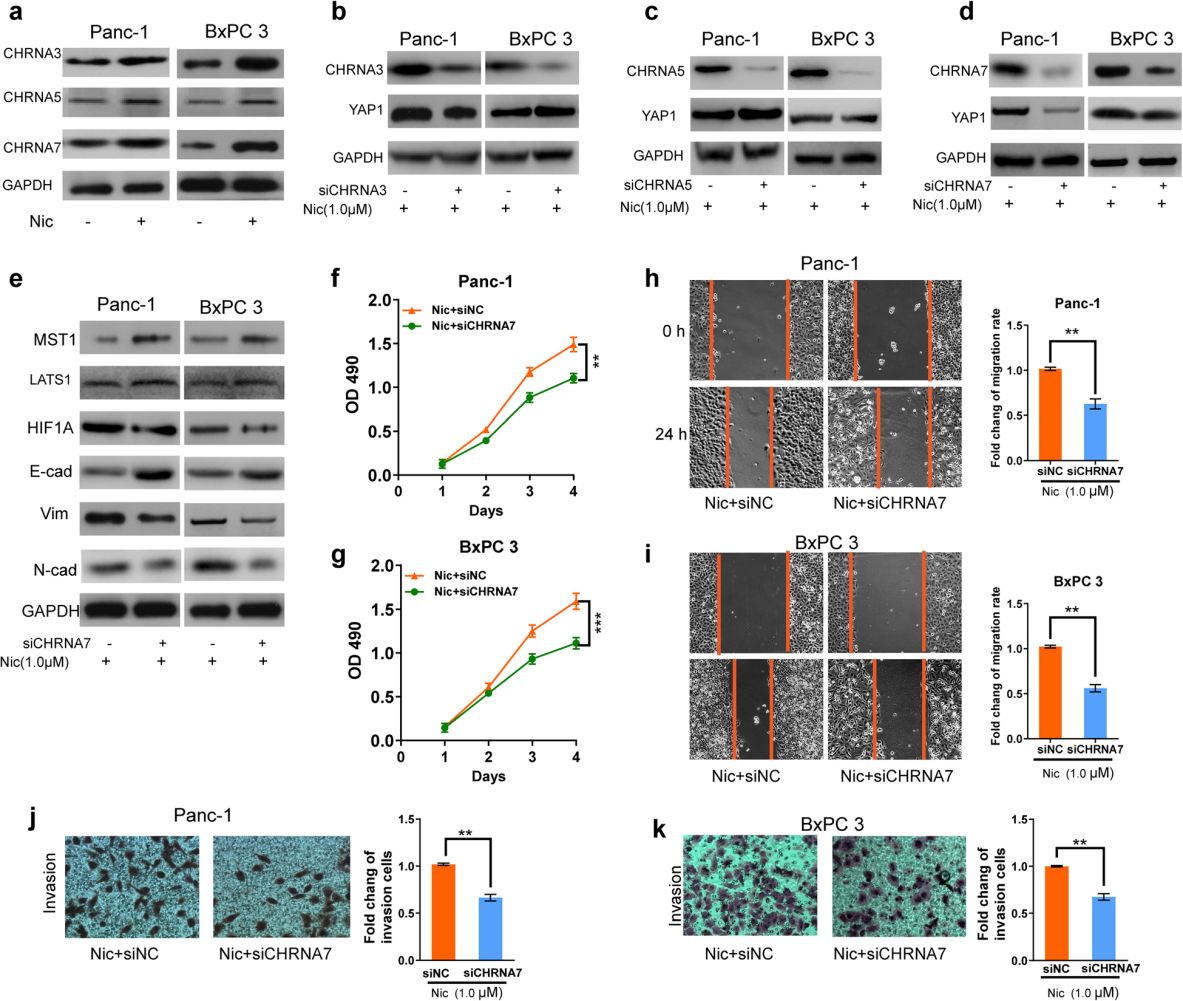
Nicotine stimulates HIF1A and YAP1 expression through association with CHRNA7. **a,** Western blotting assay of CHRNA3, CHRNA5, and CHRNA7 expression upon nicotine treatment in Panc-1 and BxPC3 cells. **b-d,** Western blotting assay of CHRNA3, 5, 7 and YAP1 expression when exposure to nicotine in Panc-1 and BxPC3 cells after transfection with siRNA targetting CHRNA3, 5, 7, respectively. **e,** Western blotting assay of HIF1A, Vim, N-cad, MST1, LATS1 and E-cad expression when exposure to nicotine in Panc-1 and BxPC3 cells after transfection with siRNA targetting CHRNA7. **f-g,** MTT assay of cell proliferation rate when exposure to nicotine in Panc-1 **(f)** and BxPC3 **(g)** cells after transfection with siRNA targetting CHRNA7. **h-i,** Wound healing assay of cell migration rate when exposure to nicotine in Panc-1 **(h)** and BxPC3 **(i)** cells after transfection with siRNA targetting CHRNA7. **j-k,** Transwell assay (with matrigel) of cell invasive potential in Panc-1 (**j**) and BxPC3 (**k**) cells after transfection with siRNA targetting CHRNA7 upon nicotine treatment. NC, negative control; CHRNA, cholinergic receptor nicotinic alpha; E-cad, E-cadherin; Vim, vimentin; N-cad, N-cadherin. GAPDH was used as an internal reference. Data are shown as the mean ± SD of three replicates.*P < 0.05; **P < 0.01; *** *P* < 0.001

Previous studies have determined that nicotine may promote cancer cell proliferation and metastasis through calcium channels or through CHRNA-3, 5, 7 in human PDAC [32, 33]. Western blot assessment confirmed that nicotine treatment activated CHRNA3, CHRNA5, and CHRNA7 (Fig. 7a). After the three receptors were silenced in Panc-1 and BxPC3 cells, respectively, we observed decreased YAP1 expression only in CHRNA7-sileneced cells following nicotine exposure (Fig. 7b-d). Additionally, we analyzed human PDAC samples from the TCGA database and found that YAP1 and HIF1A expression were significantly associated only with CHRNA7, but not with expression of CHRNA3 and CHRNA5, (Supplementary Fig. s8a-f). Furthermore, knockdown of CHRNA7 decreased the protein expression of HIF1A, Vim, and N-cad, but increased the expression of MST1, LATS1 and E-cad, in nicotine-treated PDAC cells (Fig. 7e). In addition, silencing of CHRNA7 led to the inhibition of PDAC cell proliferation, migration, and invasion (Fig. 7f-k). These results indicate that nicotine induces HIF1A and YAP1 expression through the CHRNA7 pathway.

### YAP1 enhances the stability of HIF1A protein upon nicotine treatment

**Fig. 8:**
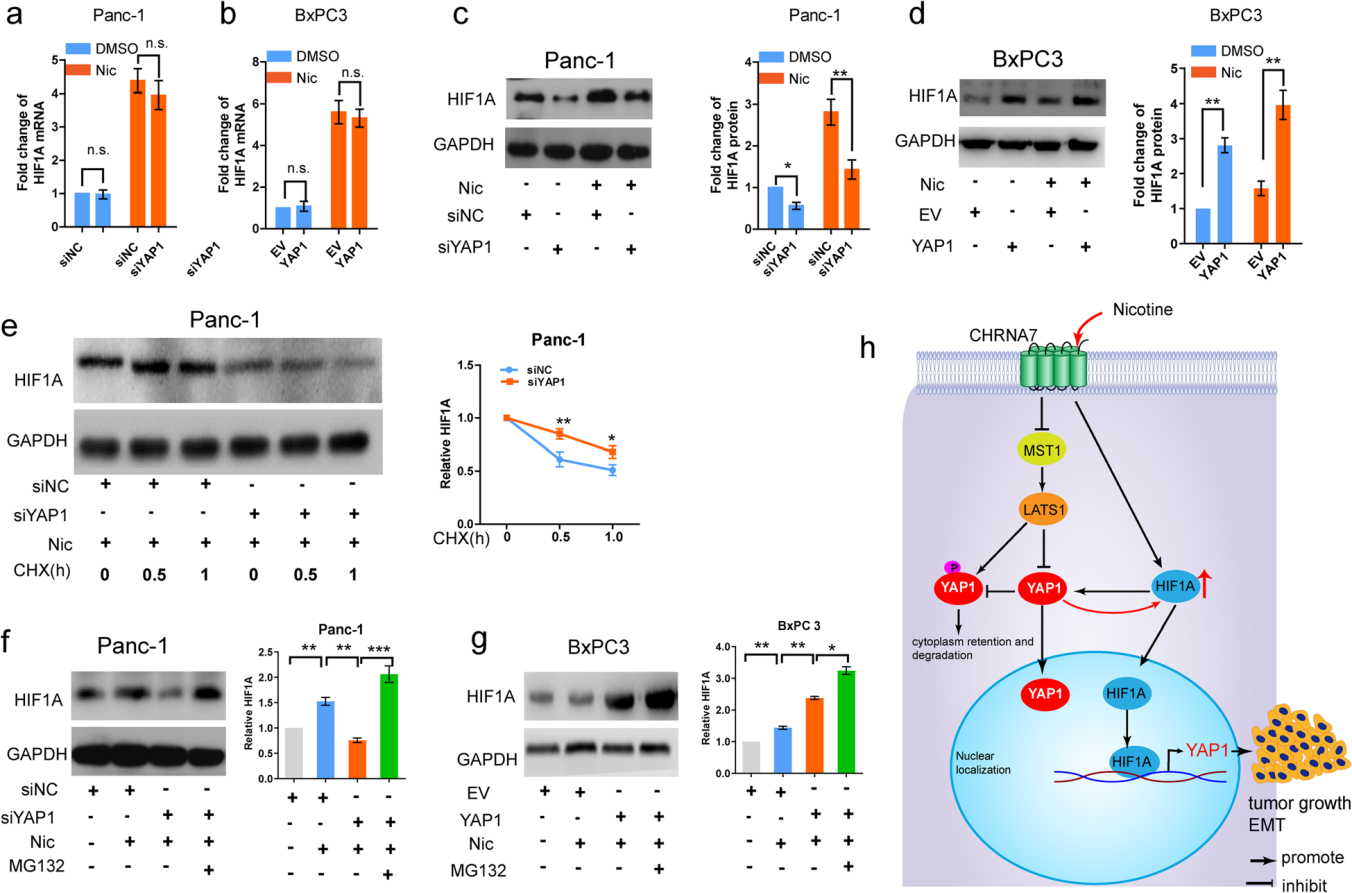
YAP1 enhances the stability of HIF1A upon nicotine treatment. **a-b,** qRT-PCR assays of the mRNA level of HIF1A in Panc-1 (**a**) and BxPC3 (**b**) cells after transfection with the indicated vectors in the absence or presence of Nic. **c-d,**, Western blot assays of the protein level of HIF1A in Panc-1 (**c**) and BxPC3 (**d**) cells after transfection with the indicated vectors in the absence or presence of Nic. **e,** After transfection with siYAP1, western blot assays of the protein level of HIF1A in cycloheximide (CHX, 100 μg/mL) treated Panc-1 cells at the different time points upon Nic treatment. **f,** After transfection with siYAP1, western blot assays of the protein level of HIF1A in MG132 (10 μM) treated Panc-1 cells upon nicotine treatment. **g,** Diagram depicts the proposed pathway involved in YAP1-mediated PDAC progression in response to nicotine. Nic, nicotine; n.s., not significant; CHRNA, cholinergic receptor nicotinic alpha. *p < 0.05, **p < 0.01

Upregulation of HIF1A protein in tumor tissues can be achieved by promoting DNA transcription, enhancing mRNA translation, or inhibiting proteasomal degradation [34]. The upregulation or knockdown of YAP1 did not change the mRNA level of HIF1A in the absence or presence of nicotine (Fig. 8a-b). We found that YAP1 knockdown substantially attenuated, while overexpression of YAP1 enhanced, expression of HIF1A protein levels in both the absence and presence of nicotine (Fig. 8c-d). Since YAP1 expression had significant effects on HIF1A protein expression, but little effect on HIF1A mRNA levels, we evaluated the effects of YAP1 on HIF1A protein in Panc-1 cells treated with cycloheximide (CHX), an inhibitor of eukaryote protein synthesis. Knockdown of endogenous YAP1 inhibited synthesis of HIF1A protein at early time points (Fig. 8e). Moreover, treatment with the proteasome inhibitor MG132 (10 μM) abolished the effects of YAP1 knockdown or overexpression (Fig. 8f-g) on HIF1A protein. These results indicated that YAP1 regulates HIF1A by enhancing the stability of the HIF1A protein.

## Discussion

Cigarette smoking is a well-established risk factor for many types of human malignancies and exerts tumorigenic effects on many cancers. We previously reported in a meta-analysis that, compared with “never smokers”, patients with PDAC who were either current or former smokers had elevated risk of total mortality [4]. Nicotine, a main active ingredient of tobacco, plays an important role in enhancing tumor cell growth, motility, and metastasis. Several studies have reported an association between nicotine and EMT induction, [35–37] but few studies [38] have examined whether and how exposure to cigarette smoke/nicotine stimulates EMT in PDAC. In the present study, we performed IHC and IF analyses, and found that smoking/nicotine exposure promoted EMT in human PDAC tissues and cell lines*.* We also found that nicotine enhanced expression levels of YAP1 and HIF1A in a dose-dependent manner, both of which induce EMT and tumor growth in PDAC cells in vitro and in murine xenograft models. Mechanistically, nicotine induces a positive feedback loop of HIF1A and YAP1, wherein HIF1A transcriptionally activates YAP1 and stimulates YAP nuclear translocation, and YAP1 increases HIF1A protein levels by enhancing the stability of HIF1A protein.

YAP1 is a key effecter of the Hippo pathway. When the Hippo pathway is activated, MST1/2 kinases phosphorylate and activate LATS1/2, which then phosphorylates YAP and TAZ (Transcriptional coActivator with a PDZ-binding motif), resulting in the cytoplasm retention and/or degradation of YAP/TAZ [16, 39]. YAP1 is also an important downstream target of KRAS signaling, and has been identified as a potential oncogene in many human tumors, including PDAC [40, 41]. Zhao et al. reported that nicotine treatment induces nuclear translocation and activation of YAP1 in ESCC, subsequently activating the PKC pathway [18]. Schaal et al. found that nicotine and e-cigarette extracts enhanced YAP1 expression via activation of CHRNA7 in NSCLC cells [19]. In line with these reports, we found that nicotine stimulated the expression of YAP1 mRNA and protein in PDAC cells. In vitro and in vivo experiments demonstrated that silencing of YAP1 inhibits nicotine-induced proliferation, migration, EMT, and expression of YAP1 target genes, while YAP1 overexpression enhanced these effects of nicotine in PDAC cells. We also found that YAP1 was more highly expressed in both TTs and ANTs of pancreas samples from patients with PDAC who were “ever smokers” compared with those who were “never smokers”. Furthermore, we demonstrated a positive correlation between high YAP1 expression and lymph node invasion and poor overall survival outcomes in PDAC. These results suggest that YAP1 is involved in smoking/nicotine exposure- induced pancreatic tumor progression.

The nuclear localization of YAP1 is a pivotal step for downstream activation of the Hippo pathway [42, 43]. A potential nuclear localization signal (NLS) was identified at the N-terminal 1–55 amino acids of Yorkie (YAP homolog in *Drosophila*) via importin alpha1 [44]. Moreover, the phosphorylation of YAP at Ser127 is a post-translational modification that enhances the cytoplasmic retention of YAP1, whereas phosphorylation of YAP1 at Ser381 by LATS1/2 induces ubiquitination and degradation [45]. In the present study, we identified nicotine as a stimulator of YAP nuclear localization, which enhanced the expression of YAP target genes (CDX2, CDC20, CTGF, and CYR61) and enhanced tumor growth. Moreover, IHC analysis of 173 pairs of human PDAC samples revealed that upregulation of YAP1 in the nucleus, but not in the cytoplasm, was significantly associated with shorter OS duration.

However, the molecular mechanisms underlying nicotine-stimulated YAP nuclear translocation and sustained activation remain unknown. A link between HIF1A expression and smoking/nicotine exposure has been identified in NSCLC and nasopharyngeal carcinoma [22, 23, 46]. Moreover, nicotine can induce nuclear accumulation of HIF1A protein, which contributes, at least in part, to nicotine-promoted NSCLC cell migration, invasion, and tumor angiogenesis [23, 46, 47]. In HCC, hypoxia was shown to promote cell survival and glycolysis by triggering YAP nuclear translocation [48, 49]. In the present research, we show, for the first time, that nicotine induced HIF1A protein accumulation in human PDAC cells. In vitro experiments revealed that overexpression of HIF1A drives YAP1 nuclear translocation and enhances YAP1 expression in PDAC cells in the presence of nicotine, whereas knockdown of HIF1α had the opposite effect. Moreover, we found a significantly positive association between YAP1 nuclear expression and HIF1A expression in human PDAC tissues. Dual luciferase assays and ChIP analyses revealed that upregulation of YAP1 in PDAC was mainly due to the transcriptional regulation by HIF1A, which bound directly to HREs on the YAP1 promoter.

Increased expression of HIF1A protein in tumor tissues can be achieved by enhancing transcription and/or mRNA translation, or by decreasing proteasomal degradation [34]. HIF1A stability is inhibited by the activation of HIF1A prolyl hydroxylases domain (PHD) and the von Hippen Lindau (VHL) tumor-suppressor protein, both of which increase HIF1A ubiquitination and degradation [50]. Our data indicate that the protein levels of HIF1A directly related to YAP1 levels, and coordinately follow the upregulation or knockdown of YAP1, although this coordinate relationship is not seen between HIF1A and YAP1 mRNA expression. Protein stability experiments involving CHX and MG132 treatment demonstrated that YAP1 enhances HIF1A levels at the post-transcriptional level by facilitating the stability of the HIF1A protein.

## Conclusions

In summary, we identified a nicotine-induced positive feedback loop between YAP1 and HIF1A, which contributes to the enhanced expression of YAP1 target genes, EMT processes, and tumorigenesis in PDAC (Fig. 8h). Developing inhibitors that specifically target YAP1 may provide a novel therapeutic approach to suppress PDAC growth, and metastasis, especially in PDAC patients who have a history of smoking.

## Supplementary information

**Additional file 1 Supplementary Table s1:** Antibodies and chemicals used in this study. **Supplementary Table s2:** The sequences of the primers included in this manuscript. **Supplementary Table s3:** Clinicopathologic characteristics of the PDAC patients from whom the TMA specimens were obtained. **Fig. S1** Smoking/nicotine exposure modulates the EMT process in PDAC cell lines and tissues. a-b, Immunohistochemical staining analysis of the expression levels of E-cad (a) and Vim (b) in TT pancreas samples from ESs and NSs (*n* = 173). Scale bar, 100 μm. c-d, Immunohistochemical stained analysis of the expression levels of E-cad (c) and Vim (d) in ANT pancreas samples from ESs and NSs. Scale bar, 100 μm. (e-f) Immunofluorescence assays of the expression levels of E-cad (e) and Vim (f) in human PDAC tissues from ESs and NSs. white scale bar, 100 μm. g, Immunofluorescence assays of the expression levels of Vim in nicotine- or DMSO-treated Panc-1 cells. h, EMT-like morphological changes of Panc-1 cells with DMSO or nicotine (1.0 μM) treatment. Scale bar, 100 μm. EMT, epithelial-mesenchymal transition; TT, tumor tissues; ANT, adjacent non-cancerous tissues; ES, ever smoker; NS, never smoker; E-cad, E-cadherin; Vim, vimentin; PDAC, pancreatic ductal adenocarcinoma; Nic, nicotine. GAPDH was used as an internal reference. Chi square test was used for statistical analysis. **Fig.S2** YAP1 mediates the effects of nicotine on the cellular functions of Panc-1 and BxPC3 cells. **a-d**, qRT-PCR and western blot assays of YAP1 expression after transfection with shYAP1 (a,b) or pcDNA4-YAP1 (c,d) and NC in Panc-1 and BxPC3 cells. **e-f,** MTT assay of cell proliferation rate in Panc-1 (e) and BxPC3 (f) cells after transfection with the indicated vectors upon nicotine treatment. g-h, Wound healing assay of cell migration rate in Panc-1 (g) and BxPC3 (h) cells after transfection with the indicated vectors upon nicotine treatment. i-j, Transwell assay (with matrigel) of cell invasive potential in Panc-1 (i) and BxPC3 (j) cells after transfection with the indicated vectors upon nicotine treatment. NC, negative control; EV, empty vector; Data are shown as the mean ± SD of three replicates.*P < 0.05; **P < 0.01; *** *P* < 0.001. **Fig. S3** Immunohistochemical staining scoring of YAP1 expression levels in TT and ANT pancreas samples according to smoking status. TT, tumor tissues; ANT, adjacent non-cancerous tissues; ES, ever smoker; NS, never smoker. Chi square test was used for statistical analysis. **Fig. S4** YAP1 expression and epithelial-mesenchymal transition markers in pancreatic ductal adenocarcinoma tissues from TCGA dataset. a, CDH1, b, Vim; c, CDH2; d, SNAI1; e, ZEB1; f, ZEB2; g, TWIST1; h, TWIST2. **Fig. S5** Immunohistochemical staining analysis of YAP1 expression patterns in PDAC from a tissue microarray (*n* = 173). a, YAP1 was weak or no staining in ANT. b-c, YAP1 was located in the nucleus (b) and cytoplasm (c). d-e, Kaplan-Meier analysis of overall survival of cases with PDAC from our cohort according to cytoplasmic (f; Log rank test, *P* = 0.183) and nuclear (f; Log rank test, *P* = 0.001) expression of YAP1. ANT, adjacent non-cancerous tissue. Scale bar, 100 μm. **Fig. S6** Semi-quantitative data of protein levels. a, Semi-quantitative data of pYAP1 protein levels in Panc-1 and BxPC3 cells after nicotine treatment. b-c, The ratio of protein abundance of pYAP1/YAP1 in Panc-1 (b) and BxPC3 (c) after nicotine treatment. d-e, The ratio of protein abundance of p-MST1/MST1 and p-LATS1/LATS1 in Panc-1 (d) and BxPC3 (e) after nicotine treatment. Protein expression levels were quantified by normalizing to the GAPDH band using image J software. *** *P* < 0.001. **Fig. S7** HIF1A mediates the effects of nicotine on the cellular functions of pancreatic ductal adenocarcinoma cells. a-d, qRT-PCR assay of HIF1A expression after transfection with shHIF1A (control: shNC, a and b) or pcDNA3.1-HIF1A (control: EV; c and d) plasmids in Panc-1 and BxPC3 cells. e-h, MTT assay of cell proliferation rate in Panc-1 and BxPC3 cells after transfection with HIF1A knockdown (e, f) and overexpression (g, h) vectors in the presence of nicotine. i-l, Wound healing assay of cell migration rate in Panc-1 and BxPC3cells after transfection with HIF1A knockdown (i, j) and overexpression (k, l) vectors in the presence of nicotine. NC, negative control; EV, empty vector; Data are shown as the mean ± SD of three replicates. **P < 0.01; *** *P <* 0.001. **Fig. S8** YAP1 and HIF1A expression and CHRNA3, 5, 7 in pancreatic ductal adenocarcinoma tissues from TCGA dataset. CHRNA, cholinergic receptor nicotinic alpha.

## Data Availability

The raw data used and analyzed in the current study are available from the corresponding author upon a reasonable request.

## Abbreviations

PDAC: Pancreatic ductal adenocarcinoma
CHRNAs: Cholinergic receptor nicotinic alphas
EMT: Epithelial-to-mesenchymal transition
E-cad: E-cadherin
Vim: Vimentin
N-cad: N-cadherin
YAP: Yes-associated protein
NSCLC: Non-small cell lung cancer
ESCC: Esophageal squamous cell cancer
HIF: Hypoxia inducible factor
HRE: Hypoxia response element
FFPE: Formalin-fixed paraffin-embedded
TT: Tumor tissue
ANT: Adjacent non-cancerous tissue
TMA: Tissue microarray
AJCC: American Joint Committee on Cancer
IHC: Immunohistochemistry
IF: Immunofluorescence
NC: Negative control
ChIP: Chromatin immunoprecipitation
OS: Overall survival
TCGA: The Cancer Genome Atlas
HR: Hazard ratio
CI: Confidence interval
PHD: Prolyl hydroxylases domain
VHL: Von Hippen Lindau

## References

[1] Bray F, Ferlay J, Soerjomataram I, Siegel RL, Torre LA, Jemal A (2018). Global cancer statistics 2018: GLOBOCAN estimates of incidence and mortality worldwide for 36 cancers in 185 countries. CA Cancer J Clin.

[2] Strobel O, Neoptolemos J, Jager D, Buchler MW (2019). Optimizing the outcomes of pancreatic cancer surgery. Nat Rev Clin Oncol.

[3] Maisonneuve P, Lowenfels AB (2015). Risk factors for pancreatic cancer: a summary review of meta-analytical studies. Int J Epidemiol.

[4] Ben QW, Liu J, Sun YW, Wang LF, Zou DW, Yuan YZ (2019). Cigarette smoking and mortality in patients with pancreatic Cancer: a systematic review and meta-analysis. Pancreas.

[5] Delitto D, Zhang D, Han S, Black BS, Knowlton AE, Vlada AC (2016). Nicotine reduces survival via augmentation of paracrine HGF-MET signaling in the pancreatic Cancer microenvironment. Clin Cancer Res.

[6] Hermann PC, Sancho P, Canamero M, Martinelli P, Madriles F, Michl P (2014). Nicotine promotes initiation and progression of KRAS-induced pancreatic cancer via Gata6-dependent dedifferentiation of acinar cells in mice. Gastroenterology.

[7] Nimmakayala RK, Seshacharyulu P, Lakshmanan I, Rachagani S, Chugh S, Karmakar S (2018). Cigarette smoke induces stem cell features of pancreatic Cancer cells via PAF1. Gastroenterology.

[8] Lamouille S, Xu J, Derynck R (2014). Molecular mechanisms of epithelial-mesenchymal transition. Nat Rev Mol Cell Biol.

[9] Nieto MA, Huang RY, Jackson RA, Thiery JP (2016). Emt: 2016. Cell.

[10] Dongre A, Weinberg RA (2019). New insights into the mechanisms of epithelial-mesenchymal transition and implications for cancer. Nat Rev Mol Cell Biol.

[11] Zhang N, Zhu T, Yu K, Shi M, Wang X, Wang L (2019). Elevation of O-GlcNAc and GFAT expression by nicotine exposure promotes epithelial-mesenchymal transition and invasion in breast cancer cells. Cell Death Dis.

[12] Vu T, Jin L, Datta PK. Effect of cigarette smoking on epithelial to Mesenchymal transition (EMT) in lung Cancer. J Clin Med. 2016;5:44. 10.3390/jcm5040044 PMC4850467 27077888

[13] Zhang L, Song X, Li X, Wu C, Jiang J (2018). Yes-associated protein 1 as a novel prognostic biomarker for gastrointestinal Cancer: a meta-analysis. Biomed Res Int.

[14] Yoo W, Lee J, Jun E, Noh KH, Lee S, Jung D, et al. The YAP1-NMU Axis is associated with pancreatic Cancer progression and poor outcome: identification of a novel diagnostic biomarker and therapeutic target. Cancers (Basel). 2019;11:1477. 10.3390/cancers11101477 PMC6826421 31575084

[15] Zhang J, Ji JY, Yu M, Overholtzer M, Smolen GA, Wang R (2009). YAP-dependent induction of amphiregulin identifies a non-cell-autonomous component of the hippo pathway. Nat Cell Biol.

[16] Rozengurt E, Sinnett-Smith J, Eibl G (2018). Yes-associated protein (YAP) in pancreatic cancer: at the epicenter of a targetable signaling network associated with patient survival. Signal Transduct Target Ther.

[17] Fang L, Teng H, Wang Y, Liao G, Weng L, Li Y (2018). SET1A-mediated mono-methylation at K342 regulates YAP activation by blocking its nuclear export and promotes tumorigenesis. Cancer Cell.

[18] Zhao Y, Zhou W, Xue L, Zhang W, Zhan Q (2014). Nicotine activates YAP1 through nAChRs mediated signaling in esophageal squamous cell cancer (ESCC). PLoS One.

[19] Schaal CM, Bora-Singhal N, Kumar DM, Chellappan SP (2018). Regulation of Sox2 and stemness by nicotine and electronic-cigarettes in non-small cell lung cancer. Mol Cancer.

[20] Butturini E. Carcereri de Prati a, Boriero D, Mariotto S. Tumor dormancy and interplay with hypoxic tumor microenvironment. Int J Mol Sci. 2019;20:4305. 10.3390/ijms20174305 PMC6747268 31484342

[21] Schito L (2018). Bridging angiogenesis and immune evasion in the hypoxic tumor microenvironment. Am J Phys Regul Integr Comp Phys.

[22] Shi D, Guo W, Chen W, Fu L, Wang J, Tian Y (2012). Nicotine promotes proliferation of human nasopharyngeal carcinoma cells by regulating alpha7AChR, ERK, HIF-1alpha and VEGF/PEDF signaling. PLoS One.

[23] Daijo H, Hoshino Y, Kai S, Suzuki K, Nishi K, Matsuo Y (2016). Cigarette smoke reversibly activates hypoxia-inducible factor 1 in a reactive oxygen species-dependent manner. Sci Rep.

[24] Edge, Byrd DR, Compton CC, Fritz AG, Greene FL, A. T (2010). AJCC Cancer Staging Handbook.

[25] Bachmann IM, Halvorsen OJ, Collett K, Stefansson IM, Straume O, Haukaas SA (2006). EZH2 expression is associated with high proliferation rate and aggressive tumor subgroups in cutaneous melanoma and cancers of the endometrium, prostate, and breast. J Clin Oncol.

[26] Xu X, Han K, Tang X, Zeng Y, Lin X, Zhao Y (2016). The ring finger protein RNF6 induces leukemia cell proliferation as a direct target of pre-B-cell leukemia Homeobox 1. J Biol Chem.

[27] Ross J, Bottardi S, Bourgoin V, Wollenschlaeger A, Drobetsky E, Trudel M (2009). Differential requirement of a distal regulatory region for pre-initiation complex formation at globin gene promoters. Nucleic Acids Res.

[28] Tang Z, Li C, Kang B, Gao G, Li C, Zhang Z (2017). GEPIA: a web server for cancer and normal gene expression profiling and interactive analyses. Nucleic Acids Res.

[29] Li JH, Liu S, Zhou H, Qu LH, Yang JH (2014). starBase v2.0: decoding miRNA-ceRNA, miRNA-ncRNA and protein-RNA interaction networks from large-scale CLIP-Seq data. Nucleic Acids Res.

[30] Ben Q, Sun Y, Liu J, Wang W, Zou D, Yuan Y. Nicotine promotes tumor progression and epithelial-mesenchymal transition by regulating the miR-155-5p/NDFIP1 axis in pancreatic ductal adenocarcinoma. Pancreatology. 2020;20:698–708. 10.1016/j.pan.2020.04.004 32354626

[31] Zanconato F, Cordenonsi M, Piccolo S (2016). YAP/TAZ at the roots of Cancer. Cancer Cell.

[32] Schaal C, Padmanabhan J, Chellappan S (2015). The role of nAChR and calcium signaling in pancreatic Cancer initiation and progression. Cancers (Basel).

[33] Al-Wadei MH, Al-Wadei HA, Schuller HM (2012). Pancreatic cancer cells and normal pancreatic duct epithelial cells express an autocrine catecholamine loop that is activated by nicotinic acetylcholine receptors alpha3, alpha5, and alpha7. Mol Cancer Res.

[34] Huang C, Li Y, Li Z, Xu Y, Li N, Ge Y (2019). LIMS1 promotes pancreatic Cancer cell survival under oxygen-glucose deprivation conditions by enhancing HIF1A protein translation. Clin Cancer Res.

[35] Wang S, Huang S, Sun YL (2017). Epithelial-Mesenchymal transition in pancreatic Cancer: a review. Biomed Res Int.

[36] Chen Y, Liu Z, Wang H, Tang Z, Liu Y, Liang Z (2020). VPS33B negatively modulated by nicotine functions as a tumor suppressor in colorectal cancer. Int J Cancer.

[37] Kim SM, Hwang KA, Choi DW, Choi KC (2018). The cigarette smoke components induced the cell proliferation and epithelial to mesenchymal transition via production of reactive oxygen species in endometrial adenocarcinoma cells. Food Chem Toxicol.

[38] Dasgupta P, Rizwani W, Pillai S, Kinkade R, Kovacs M, Rastogi S (2009). Nicotine induces cell proliferation, invasion and epithelial-mesenchymal transition in a variety of human cancer cell lines. Int J Cancer.

[39] Panciera T, Azzolin L, Cordenonsi M, Piccolo S (2017). Mechanobiology of YAP and TAZ in physiology and disease. Nat Rev Mol Cell Biol.

[40] Murakami S, Nemazanyy I, White SM, Chen H, Nguyen CDK, Graham GT (2019). A yap-Myc-Sox2-p53 regulatory network dictates metabolic homeostasis and differentiation in Kras-driven pancreatic ductal adenocarcinomas. Dev Cell.

[41] Eibl G, Rozengurt E (2019). KRAS, YAP, and obesity in pancreatic cancer: a signaling network with multiple loops. Semin Cancer Biol.

[42] Shen Y, Zhao S, Wang S, Pan X, Zhang Y, Xu J (2019). S1P/S1PR3 axis promotes aerobic glycolysis by YAP/c-MYC/PGAM1 axis in osteosarcoma. EBioMedicine.

[43] Yao F, Zhou Z, Kim J, Hang Q, Xiao Z, Ton BN (2018). SKP2- and OTUD1-regulated non-proteolytic ubiquitination of YAP promotes YAP nuclear localization and activity. Nat Commun.

[44] Guo H, Liu J, Ben Q, Qu Y, Li M, Wang Y (2016). The aspirin-induced long non-coding RNA OLA1P2 blocks phosphorylated STAT3 homodimer formation. Genome Biol.

[45] Zhao B, Li L, Tumaneng K, Wang CY, Guan KL (2010). A coordinated phosphorylation by Lats and CK1 regulates YAP stability through SCF (beta-TRCP). Genes Dev.

[46] Wang Y, He J, Jiang H, Zhang Q, Yang H, Xu X (2018). Nicotine enhances storeoperated calcium entry by upregulating HIF1alpha and SOCC components in nonsmall cell lung cancer cells. Oncol Rep.

[47] Zhang Q, Tang X, Zhang ZF, Velikina R, Shi S, Le AD (2007). Nicotine induces hypoxia-inducible factor-1alpha expression in human lung cancer cells via nicotinic acetylcholine receptor-mediated signaling pathways. Clin Cancer Res.

[48] Zhou TY, Zhuang LH, Hu Y, Zhou YL, Lin WK, Wang DD (2016). Inactivation of hypoxia-induced YAP by statins overcomes hypoxic resistance tosorafenib in hepatocellular carcinoma cells. Sci Rep.

[49] Zhang X, Li Y, Ma Y, Yang L, Wang T, Meng X (2018). Yes-associated protein (YAP) binds to HIF-1alpha and sustains HIF-1alpha protein stability to promote hepatocellular carcinoma cell glycolysis under hypoxic stress. J Exp Clin Cancer Res.

[50] Tan Z, Xu J, Zhang B, Shi S, Yu X, Liang C. Hypoxia: a barricade to conquer the pancreatic cancer. Cell Mol Life Sci. 2020;77:3077–83. 10.1007/s00018-019-03444-3 PMC11104901 31907561

