# Supplementary material for: A nicotine-induced positive feedback loop between HIF1A and YAP1 contributes to epithelial-to-mesenchymal transition in pancreatic ductal adenocarcinoma
Source: J Exp Clin Cancer Res. 2020 Sep 7;39:181. doi: 10.1186/s13046-020-01689-6 (PMC7487530; doi:10.1186/s13046-020-01689-6)

**A nicotine-induced positive feedback loop between HIF1A and YAP1 contributes to epithelial-to-mesenchymal transition in pancreatic ductal adenocarcinoma**

Qiwen Ben^1^, Wei An^2^, Yunwei Sun^1^, Jun Liu^3^, Duowu Zou^1^, Yaozong Yuan^1^

1 Department of Gastroenterology, Ruijin Hospital, Shanghai Jiaotong University School of Medicine, Shanghai, PR China

2 Wei An, Department of Gastroenterology, Changhai Hospital of Second Military Medical University, 168 Changhai Road, Shanghai 200433, China.

3 Department of Oncology, Shanghai General Hospital, Shanghai Jiao Tong University School of Medicine, Shanghai, PR China

**Supplementary** Table s1: Antibodies and chemicals used in this study

| **Antibodies** | **Company** | **Product code** |
| --- | --- | --- |
| Rabbit mAb YAP1 | CST | #14074 |
| Rabbit mAb p-YAP1(Ser127) | CST | #13008 |
| LATS1 (C66B5) | CST | #3477 |
| CHRNA3 | Santa Cruz | sc-365479 |
| CHRNA5 | Santa Cruz | sc-376979 |
| CHRNA7 | Santa Cruz | sc-58607 |
| Mouse mAb HIF1A | abcam | Ab2185 |
| Rabbit mAb E-cadherin | CST | #3195 |
| Rabbit mAb Vimentin (D21H3) | CST | #5741 |
| N-cadherin | abcam | ab98952 |
| Rabbit mAb MST1 | CST | #3682 |
| p-MST1(Thr183) | CST | #49332 |
| p-LATS1 (Thr1079) | CST | #8654 |
| Claudin-1 | abcam | ab15098 |
| Cycloheximide | MedChem Express | HY-12320 |
| MG132 | MedChem Express | HY-13259 |
| Nicotine | Sigma | BP822 |

**Supplementary Table s2:** The sequences of the primers included in this manuscript

|  | Forward premier (5’-3’) | Reverse premier (5’-3’) |
| --- | --- | --- |
| **Primers for qRT-PCR** | | |
| E-cadherin | CGAGAGCTACACGTTCACGG | GGGTGTCGAGGGAAAAATAGG |
| Vimentin | TGCCGTTGAAGCTGCTAACTA | CCAGAGGGAGTGAATCCAGATTA |
| β-actin | GATCATTGCTCCTCCTGAGC | ACTCCTGCTTGCTGATCCAC |
| YAP1 | CCTTCTTCAAGCCGCCGGAG | CAGTGTCCCAGGAGAAACAGC |
| HIF1A | CGTGTTATCTGTCGCTTTGAGTC | GTCTGGCTGCTGTAATAATGTTCC |
| GAPDH | GGTGAAGGTCGGAGTCAACGG | GAGGTCAATGAAGGGGTCATTG |
| CHRNA7 | GCTGCTCGTGGCTGAGATC | TGGCGAAGTACTGGGCTATCA |
| h CDX2 | GACGTGAGCATGTACCCTAGC | GCGTAGCCATTCCAGTCCT |
| h CYR61 | AGCCTCGCATCCTATACAACC | TTCTTTCACAAGGCGGCACTC |
| h CTGF | AAAAGTGCATCCGTACTCCCA | CCGTCGGTACATACTCCACAG |
| h CDC20 | GCACAGTTCGCGTTCGAGA | CTGGATTTGCCAGGAGTTCGG |
| m CDX2 | CAAGGACGTGAGCATGTATCC | GTAACCACCGTAGTCCGGGTA |
| m CYR61 | CTGCGCTAAACAACTCAACGA | GCAGATCCCTTTCAGAGCGG |
| m CTGF | GGGCCTCTTCTGCGATTTC | ATCCAGGCAAGTGCATTGGTA |
| m CDC20 | GTTCGTGTTCGAGAGCGATTT | CTAGGGGTGGTCTGAACCTT |
| **Primers for YAP1 promoter constructs** | | |
| HRE1 | TGCTCTAGTAAAGGGACAGCT | ATCACTTGGTGTGGTAGGGC |
| HRE2 | GAAGGGACTACCAACAGGGT | ACGGTGGACTCCGTTAATGT |
| HRE3 | ATGCATAGTTTCTGCCCAAAGG | CCCTGTTGGTAGTCCCTTCC |
| Primer for ChIP | | |
|  | GCATAGTTTCTGCCCAAAGGT | AATGAATGGGCAAGGAGGTCA |
| **siRNA targeting sequence** | | |
| HIF1A#1 | CGGCGAAGTAAAGAATCTGAA |  |
| HIF1A# 2 | TGATGAAAGAATTACCGAATT |  |
| YAP1#1 | 5’-GGTGATACTATCAACCAAA-3’ |  |
| YAP1#2 | 5’-CACATTAACGACTAGATTA-3’ |  |

**Supplementary Table s3:** Clinicopathologic characteristics of the PDAC patients from whom the TMA specimens were obtained

| **Parameters** | **Total**  **(n=173)** | **%** |
| --- | --- | --- |
| **Median age, years** | 59 |  |
| range | 28-81 |  |
| **Gender** |  |  |
| Male | 108 | 62.4 |
| Female | 65 | 37.6 |
| **Tumor localization** |  |  |
| Head | 111 | 64.2 |
| Others | 62 | 35.8 |
| **CA199,u/L** |  | 0.0 |
| ＜37 | 51 | 29.5 |
| ≥37 | 122 | 70.5 |
| **Tumor size, cm** |  |  |
| ＜4 | 97 | 56.1 |
| ≥4 | 76 | 43.9 |
| **T stage** |  |  |
| T1 | 2 | 1.2 |
| **T2** | 138 | 79.8 |
| **T3** | 28 | 16.2 |
| T4 | 5 | 2.9 |
| **N stage** |  |  |
| N0 | 109 | 63.0 |
| N1 | 64 | 37.0 |
| **Tumor grade** |  |  |
| Poor | 28 | 16.2 |
| Moderate | 126 | 72.8 |
| Well | 19 | 11.0 |

**Supple. Figure legends**

**Fig.s1.** Smoking/nicotine exposure modulates the EMT process in PDAC cell lines and tissues

a-b, Immunohistochemical staining analysis of the expression levels of E-cad (a) and Vim (b) in TT pancreas samples from ESs and NSs (n=173). Scale bar, 100μm. c-d, Immunohistochemical stained analysis of the expression levels of E-cad (c) and Vim (d) in ANT pancreas samples from ESs and NSs. Scale bar, 100μm. (e-f) Immunofluorescence assays of the expression levels of E-cad (e) and Vim (f) in human PDAC tissues from ESs and NSs. white scale bar, 100μm. g, Immunofluorescence assays of the expression levels of Vim in nicotine- or DMSO-treated Panc-1 cells. h, EMT-like morphological changes of Panc-1 cells with DMSO or nicotine (1.0 μM) treatment. Scale bar, 100μm. EMT, epithelial-mesenchymal transition; TT, tumor tissues; ANT, adjacent non-cancerous tissues; ES, ever smoker; NS, never smoker; E-cad, E-cadherin; Vim, vimentin; PDAC, pancreatic ductal adenocarcinoma; Nic, nicotine. GAPDH was used as an internal reference. Chi square test was used for statistical analysis.


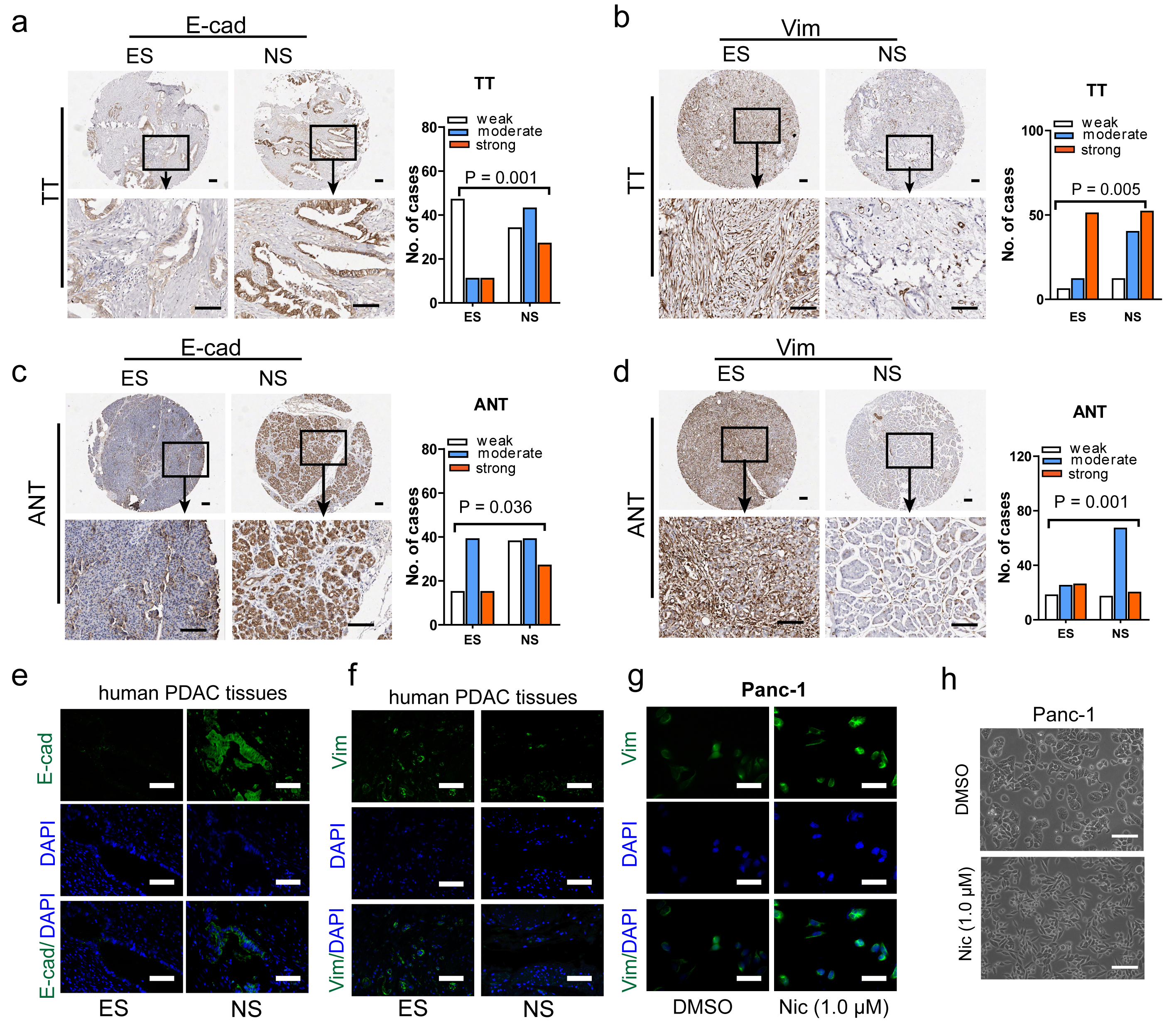


**Supple. Fig.s2**-YAP1 mediates the effects of nicotine on the cellular functions of Panc-1 and BxPC3 cells.

**a-d**, qRT-PCR and western blot assays of YAP1 expression after transfection with shYAP1 (a,b) or pcDNA4-YAP1 (c,d) and NC in Panc-1 and BxPC3 cells. **e-f,** MTT assay of cell proliferation rate in Panc-1 (e) and BxPC3 (f) cells after transfection with the indicated vectors upon nicotine treatment. g-h, Wound healing assay of cell migration rate in Panc-1 (g) and BxPC3 (h) cells after transfection with the indicated vectors upon nicotine treatment. i-j, Transwell assay (with matrigel) of cell invasive potential in Panc-1 (i) and BxPC3 (j) cells after transfection with the indicated vectors upon nicotine treatment. NC, negative control; EV, empty vector; Data are shown as the mean ± SD of three replicates.*P < 0.05; **P < 0.01; *** P < 0.001.


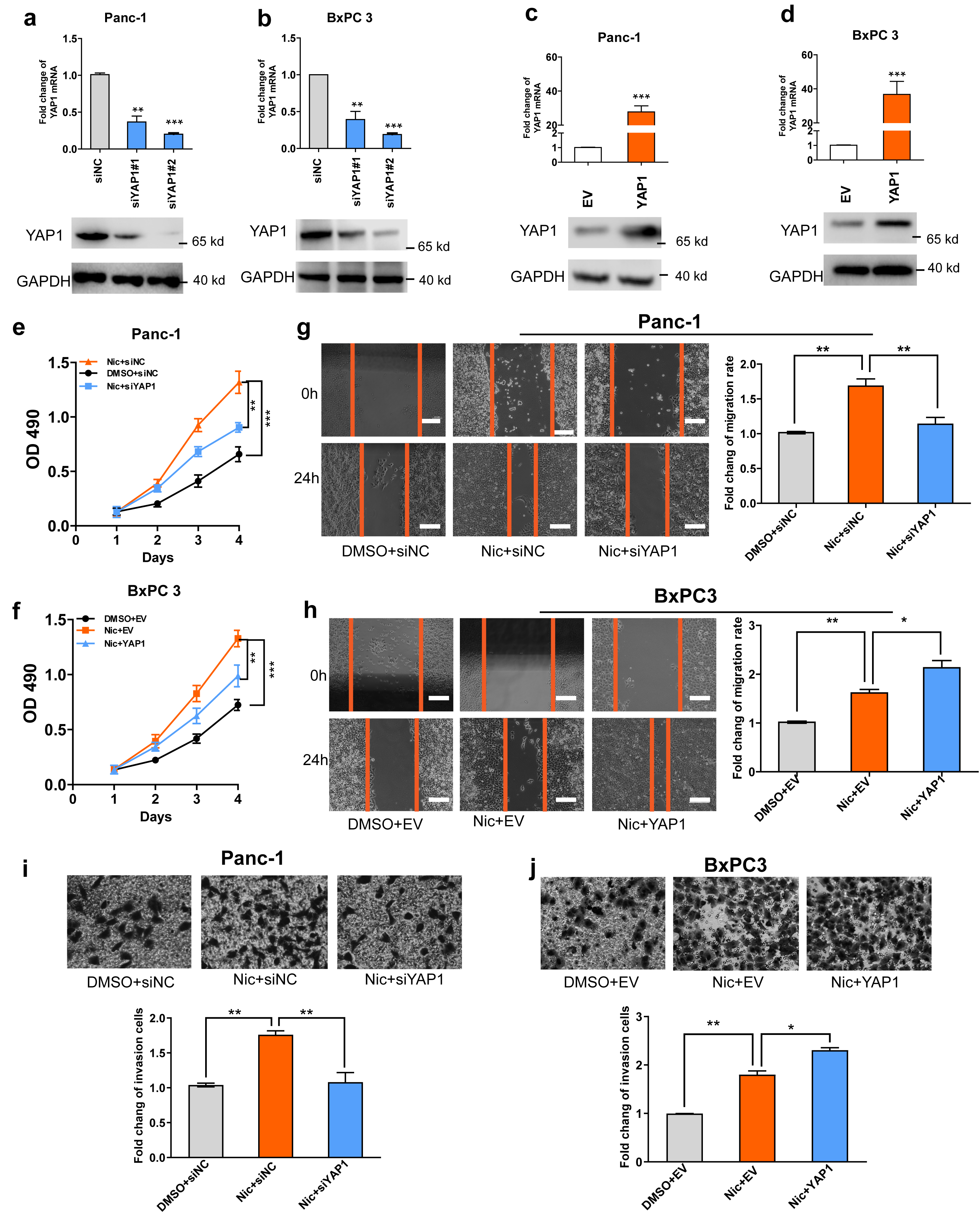


**Supple. Fig.s3-** Immunohistochemical staining scoring of YAP1 expression levels in TT and ANT pancreas samples according to smoking status. TT, tumor tissues; ANT, adjacent non-cancerous tissues; ES, ever smoker; NS, never smoker. Chi square test was used for statistical analysis.


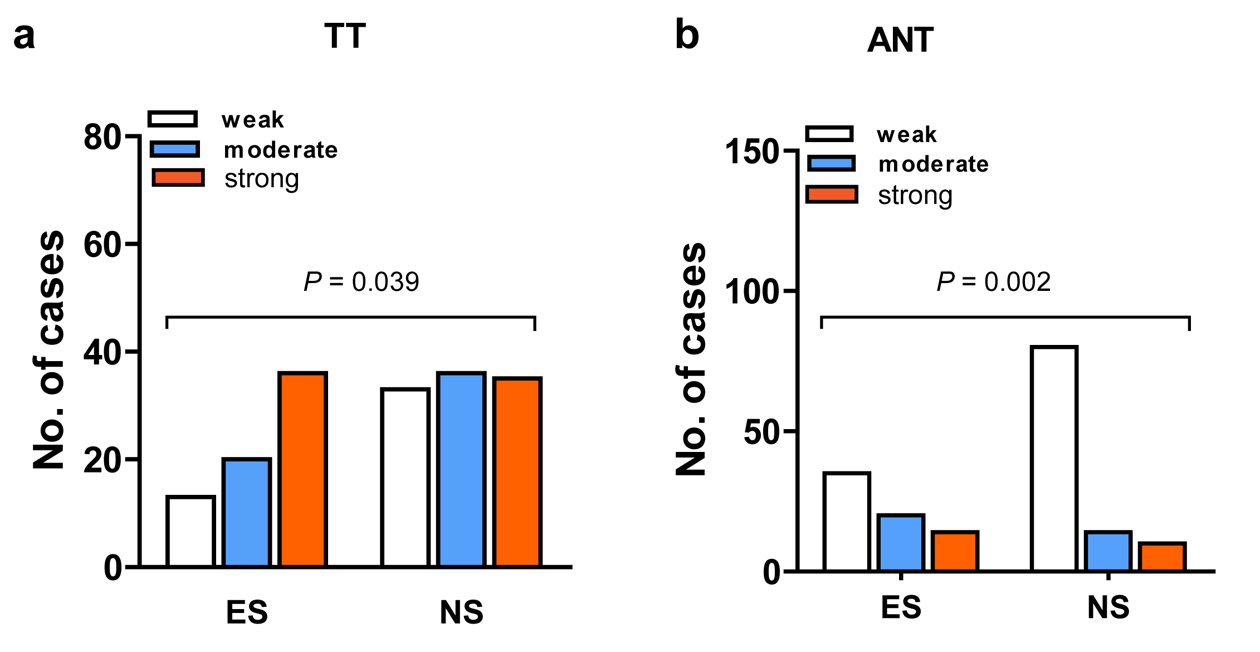


**Supple. Fig.s4-** YAP1 expression and epithelial-mesenchymal transition markers in pancreatic ductal adenocarcinoma tissues from TCGA dataset.

a, CDH1, b, Vim; c, CDH2; d, SNAI1; e, ZEB1; f, ZEB2; g, TWIST1; h, TWIST2


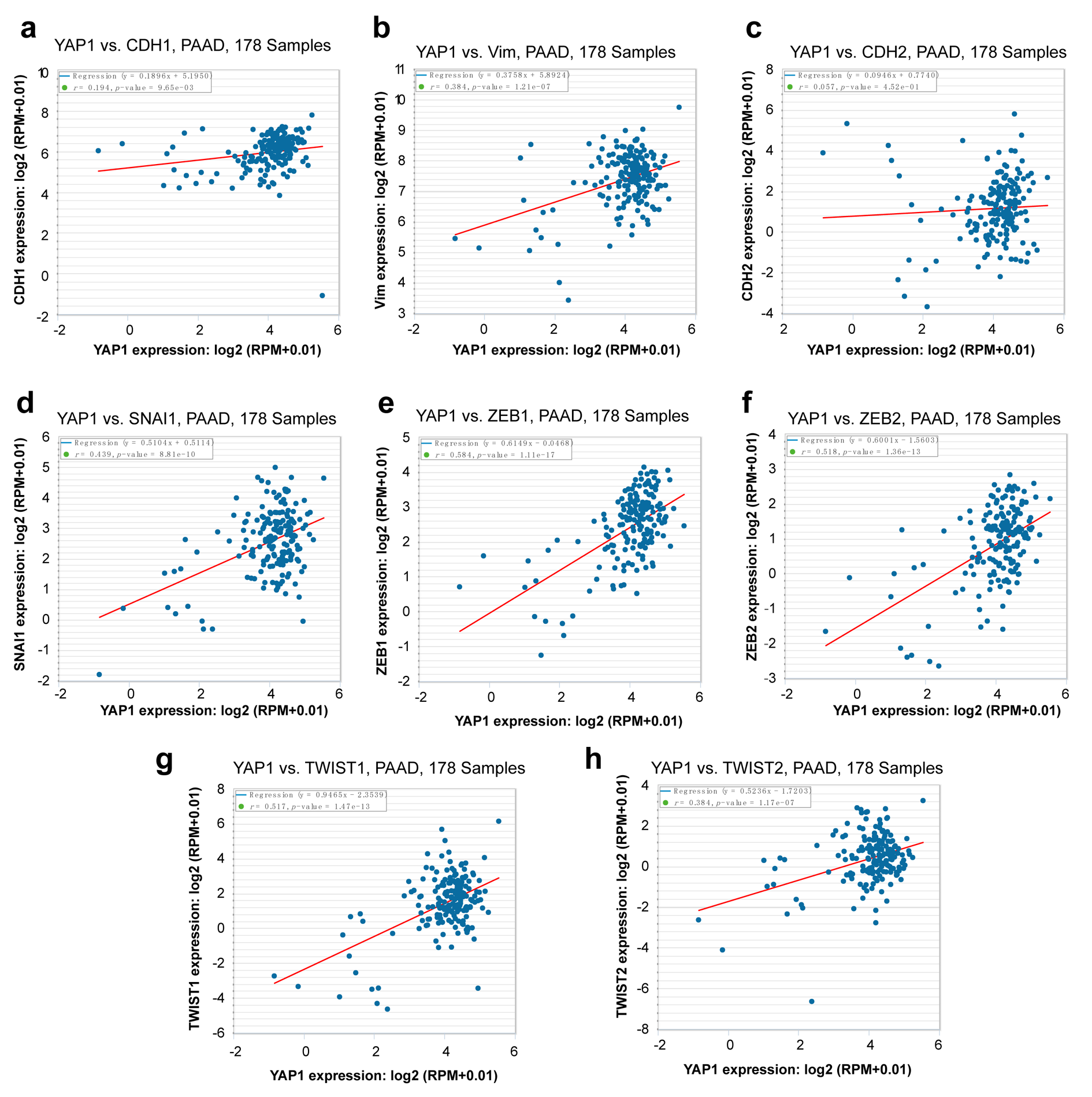


**Supple. Fig.s5-** Immunohistochemical staining analysis of YAP1 expression patterns in PDAC from a tissue microarray (n = 173). a, YAP1 was weak or no staining in ANT. b-c, YAP1 was located in the nucleus (b) and cytoplasm (c). d-e, Kaplan-Meier analysis of overall survival of cases with PDAC from our cohort according to cytoplasmic (f; Log rank test, *P* = 0.183) and nuclear (f; Log rank test, *P* = 0.001) expression of YAP1. ANT, adjacent non-cancerous tissue. Scale bar, 100μm.


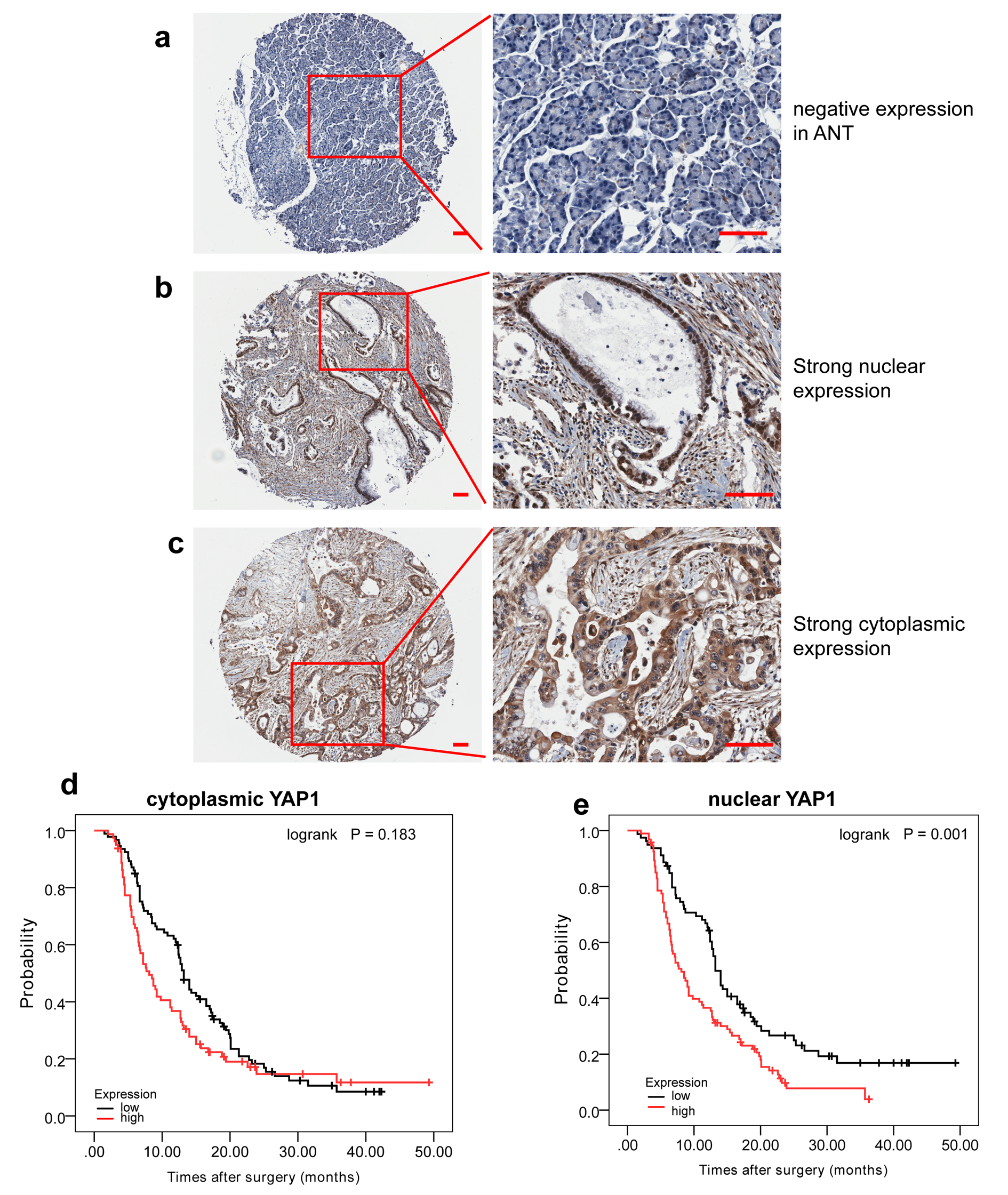


**Supple. Fig.s6-** Semi-quantitative data of protein levels. a, Semi-quantitative data of pYAP1 protein levels in Panc-1 and BxPC3 cells after nicotine treatment. b-c, The ratio of protein abundance of pYAP1/YAP1 in Panc-1 (b) and BxPC3 (c) after nicotine treatment. d-e, The ratio of protein abundance of p-MST1/MST1 and p-LATS1/LATS1 in Panc-1 (d) and BxPC3 (e) after nicotine treatment. Protein expression levels were quantified by normalizing to the GAPDH band using image J software. *** P < 0.001.

**
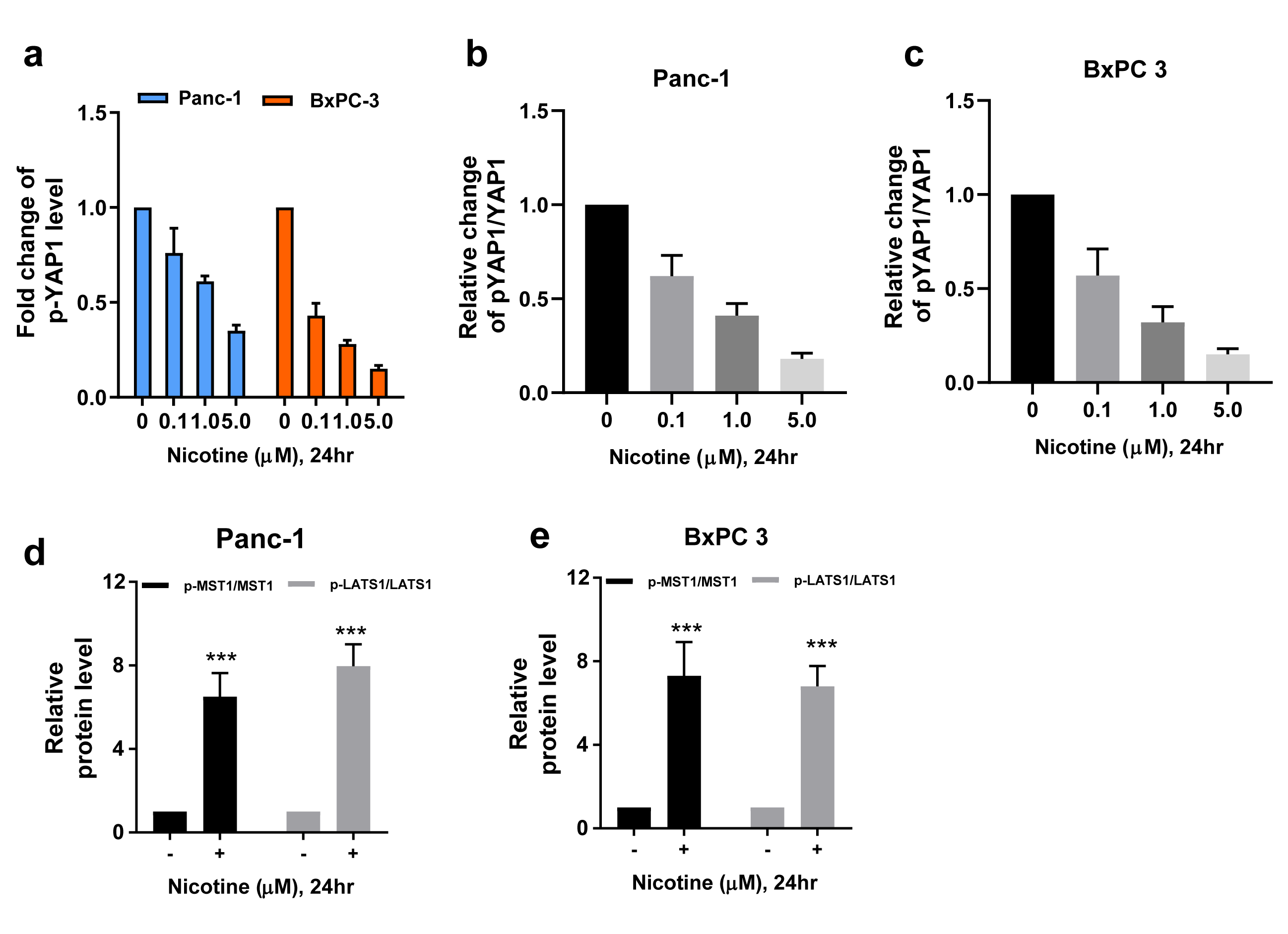
**

**Supple. Fig.s7-** HIF1A mediates the effects of nicotine on the cellular functions of pancreatic ductal adenocarcinoma cells. a-d, qRT-PCR assay of HIF1A expression after transfection with shHIF1A (control: shNC, a and b) or pcDNA3.1-HIF1A (control: EV; c and d) plasmids in Panc-1 and BxPC3 cells. e-h, MTT assay of cell proliferation rate in Panc-1 and BxPC3 cells after transfection with HIF1A knockdown (e, f) and overexpression (g, h) vectors in the presence of nicotine. i-l, Wound healing assay of cell migration rate in Panc-1 and BxPC3cells after transfection with HIF1A knockdown (i, j) and overexpression (k, l) vectors in the presence of nicotine. NC, negative control; EV, empty vector; Data are shown as the mean ± SD of three replicates. **P < 0.01; *** P < 0.001.


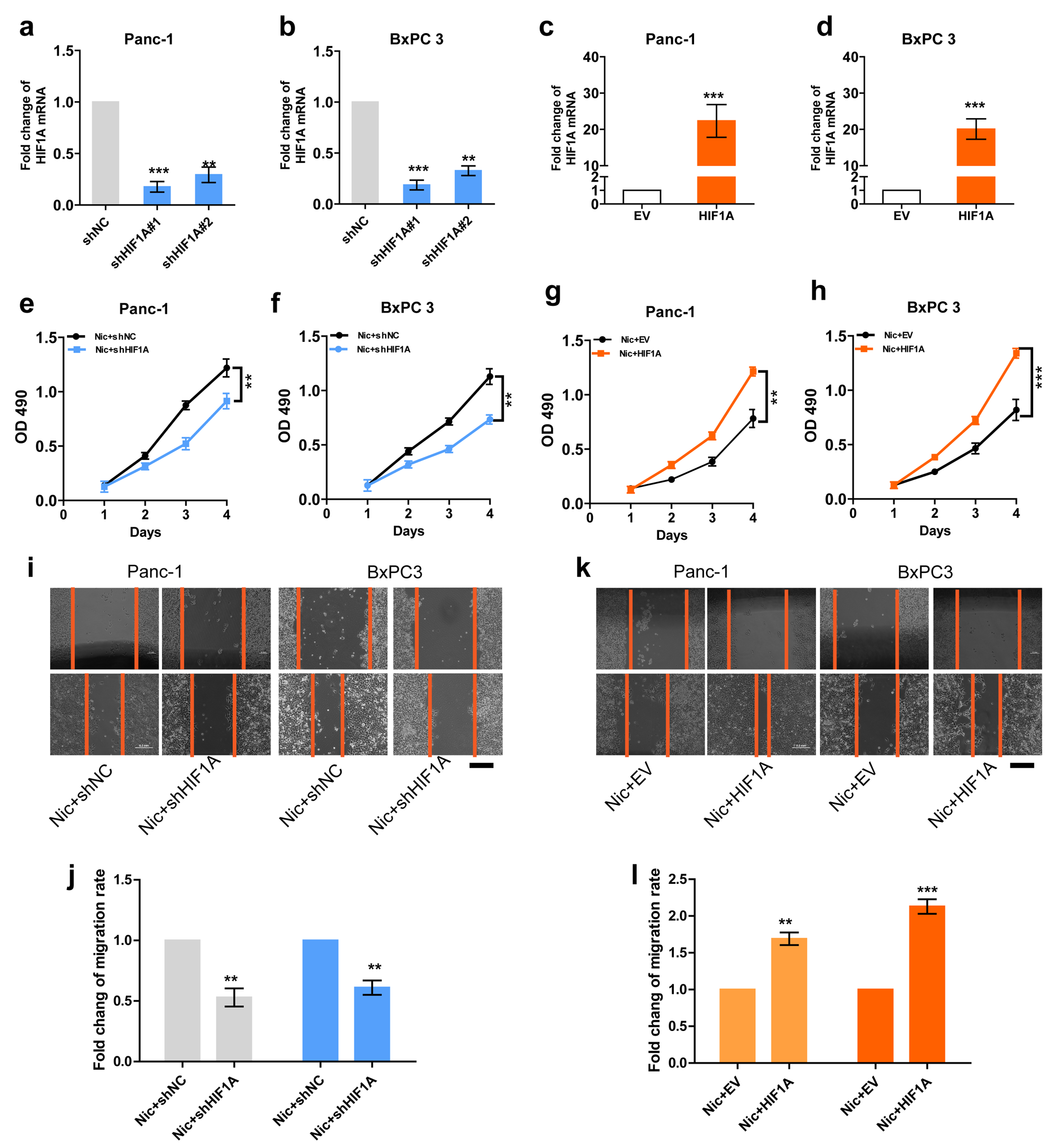


**Supple. Fig.s8-** YAP1 and HIF1A expression and CHRNA3, 5, 7 in pancreatic ductal adenocarcinoma tissues from TCGA dataset. CHRNA, cholinergic receptor nicotinic alpha.


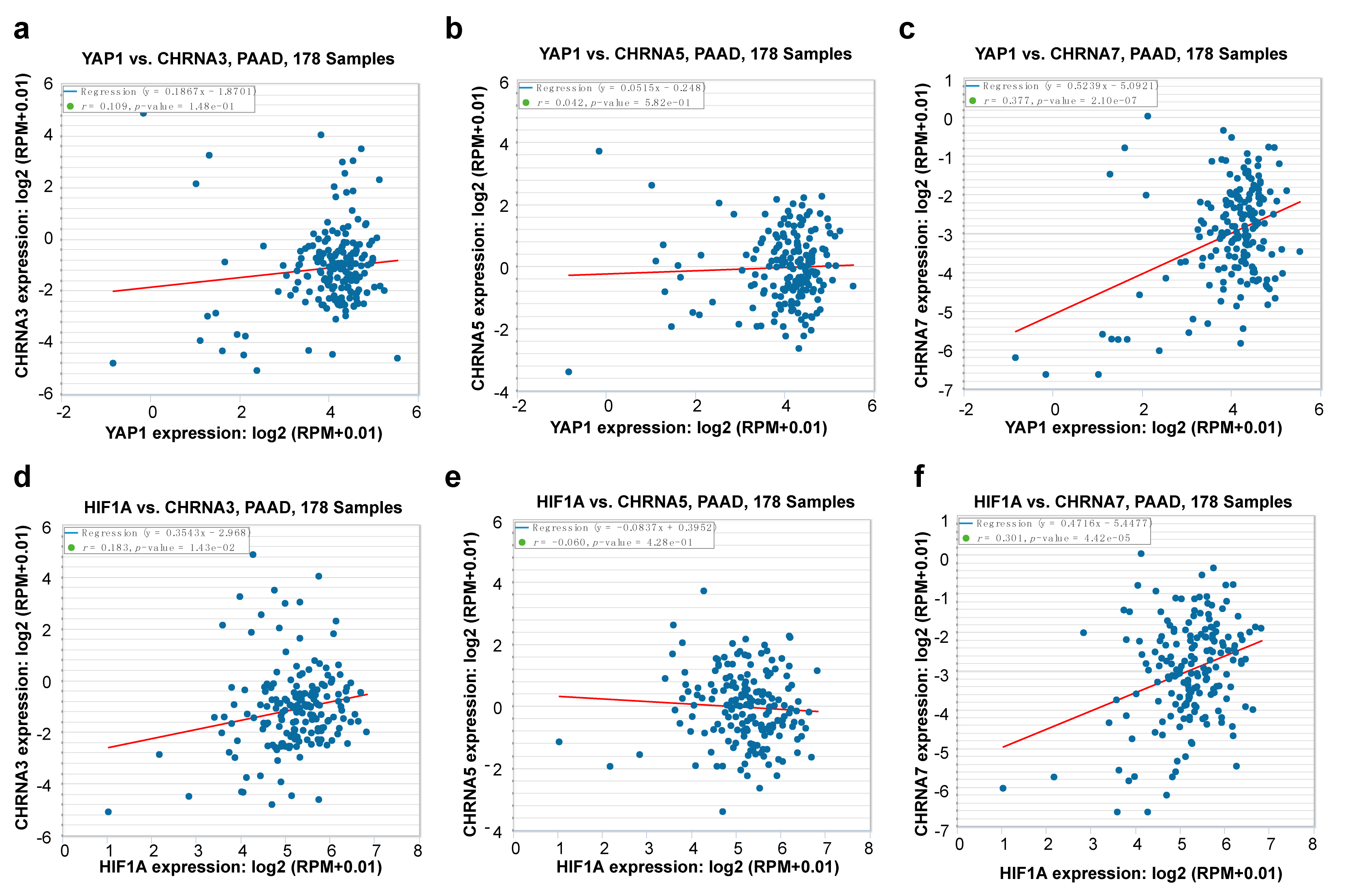

Supplement: Supplementary file 1 — Additional file 1 Supplementary Table s1: Antibodies and chemicals used in this study. Supplementary Table s2: The sequences of the primers included in this manuscript. Supplementary Table s3: Clinicopathologic characteristics of the PDAC patients from whom the TMA specimens were obtained. Fig. S1 Smoking/nicotine exposure modulates the EMT process in PDAC cell lines and tissues. a-b, Immunohistochemical staining analysis of the expression levels of E-cad (a) and Vim (b) in TT pancreas samples from ESs and NSs (n = 173). Scale bar, 100 μm. c-d, Immunohistochemical stained analysis of the expression levels of E-cad (c) and Vim (d) in ANT pancreas samples from ESs and NSs. Scale bar, 100 μm. (e-f) Immunofluorescence assays of the expression levels of E-cad (e) and Vim (f) in human PDAC tissues from ESs and NSs. white scale bar, 100 μm. g, Immunofluorescence assays of the expression levels of Vim in nicotine- or DMSO-treated Panc-1 cells. h, EMT-like morphological changes of Panc-1 cells with DMSO or nicotine (1.0 μM) treatment. Scale bar, 100 μm. EMT, epithelial-mesenchymal transition; TT, tumor tissues; ANT, adjacent non-cancerous tissues; ES, ever smoker; NS, never smoker; E-cad, E-cadherin; Vim, vimentin; PDAC, pancreatic ductal adenocarcinoma; Nic, nicotine. GAPDH was used as an internal reference. Chi square test was used for statistical analysis. Fig.S2 YAP1 mediates the effects of nicotine on the cellular functions of Panc-1 and BxPC3 cells. a-d, qRT-PCR and western blot assays of YAP1 expression after transfection with shYAP1 (a,b) or pcDNA4-YAP1 (c,d) and NC in Panc-1 and BxPC3 cells. e-f, MTT assay of cell proliferation rate in Panc-1 (e) and BxPC3 (f) cells after transfection with the indicated vectors upon nicotine treatment. g-h, Wound healing assay of cell migration rate in Panc-1 (g) and BxPC3 (h) cells after transfection with the indicated vectors upon nicotine treatment. i-j, Transwell assay (with matrigel) of cell invasive potential in Panc-1 (i) and [file 13046_2020_1689_MOESM1_ESM.doc]
